# Anti‐inflammatory mechanisms in cancer research: Characterization of a distinct M2‐like macrophage model derived from the THP‐1 cell line

**DOI:** 10.1002/cam4.6681

**Published:** 2023-11-30

**Authors:** Katharina M. Scheurlen, Dylan L. Snook, Andrew B. Littlefield, Joan B. George, Mary A. Parks, Robert J. Beal, Anne MacLeod, Daniel W. Riggs, Jeremy T. Gaskins, Julia Chariker, Eric C. Rouchka, Susan Galandiuk

**Affiliations:** ^1^ Digestive Surgery Research Laboratory, Price Institute of Surgical Research, The Hiram C. Polk, Jr, MD Department of Surgery University of Louisville School of Medicine Louisville Kentucky USA; ^2^ Christina Lee Brown Envirome Institute, Division of Environmental Medicine, Department of Medicine University of Louisville Louisville Kentucky USA; ^3^ Department of Bioinformatics & Biostatistics University of Louisville Louisville Kentucky USA; ^4^ Kentucky IDeA Networks of Biomedical Research Excellence (KY INBRE), Bioinformatics Core University of Louisville Louisville Kentucky USA

**Keywords:** colon cancer, colonic neoplasms, immunology, inflammation, RNA‐Seq, THP‐1 cells, tumor‐associated macrophages

## Abstract

**Aims:**

Macrophages play an essential role in cancer development. Tumor‐associated macrophages (TAMs) have predominantly M2‐like attributes that are associated with tumor progression and poor patient survival. Numerous methods have been reported for differentiating and polarizing macrophages in vitro, but there is no standardized and validated model for creating TAMs. Primary cells show varying cytokine responses depending on their origin and functional studies utilizing these cells may lack generalization and validity. A distinct cell line‐derived TAM‐like M2 subtype is required to investigate the mechanisms mediated by anti‐inflammatory TAMs in vitro. Our previous work demonstrated a standardized protocol for creating an M2 subtype derived from a human THP‐1 cell line. The cell expression profile, however, has not been validated. The aim of this study was to characterize and validate the TAM‐like M2 subtype macrophage created based on our protocol to introduce them as a standardized model for cancer research.

**Methods and results:**

Using qRT‐PCR and ELISA, we demonstrated that proinflammatory, anti‐inflammatory, and tumor‐associated marker expression changed during THP‐1‐derived marcrophage development in vitro, mimicking a TAM‐related profile (e.g., TNFα, IL‐1β). The anti‐inflammatory marker IL‐8/CXCL8, however, is most highly expressed in young M0 macrophages. Flow cytometry showed increased expression of CD206 in the final TAM‐like M2 macrophage. Single‐cell RNA‐sequencing analysis of primary human monocytes and colon cancer tissue macrophages demonstrated that cell line‐derived M2 macrophages resembled a TAM‐related gene profile.

**Conclusions:**

The THP‐1‐derived M2 macrophage based on a standardized cell line model represents a distinct anti‐inflammatory TAM‐like phenotype with an M2a subtype profile. This model may provide a basis for in vitro investigation of functional mechanisms in a variety of anti‐inflammatory settings, particularly colon cancer development.

## 1 INTRODUCTION

Macrophages have various roles in cancer development, including inflammation, proliferation, and tissue remodeling.^1^, ^2^ Tissue macrophages induce responses that can have either local or systemic effects, ranging from the regulation of wound healing to the induction of acute phase protein synthesis through hepatocytes, thereby affecting systemic inflammation and metabolism.^2^, ^3^

Numerous in vitro models have been reported, using either primary human monocytes or cell line‐derived macrophages.^4^, ^5^, ^6^, ^7^, ^8^ These models show varying cytokine responses depending on their origin and the respective cell treatment, which makes comparing findings of different studies difficult. A standardized approach using a cell line model with reproducible cellular responses mimicking primary human cells is needed to gain insight into the complexity of macrophage‐mediated cancer development.

Two major subtypes of macrophages can be defined depending on their origin. Contrary to the erroneous assumption that tissue macrophages mostly originate from circulating bone marrow monocytes that invade tissues, most local macrophage populations are derived from embryonic progenitors that are seeded before birth.^1^, ^9^, ^10^ These fetal macrophages initially evolve from yolk sac blood islands that develop during the first 2 weeks of gestation, and in adults they are believed to maintain their population by self‐renewal.^11^ Whether they can actually persist through adulthood and to which extent they replenish their populations is currently still discussed.^12^

In contrast, bone marrow‐derived tissue macrophages are part of the well‐established mononuclear phagocytic system (MPS) and evolve from circulating peripheral monocytes.^9^ Among the mononuclear phagocytic lineage, macrophages represent the final cell type after differentiation of monocytes that originate in the bone marrow.^9^

The monocyte‐like THP‐1 cell line was derived from peripheral monocytes of a 1‐year‐old infant with acute monocytic leukemia and has been widely used as an in vitro model for demonstrating mechanisms in human monocytes and macrophages.^13^, ^14^ THP‐1 cells can be differentiated into macrophage‐like cells and then polarized into either proinflammatory M1 or anti‐inflammatory M2 phenotypes. Due to their high plasticity, macrophages can continuously switch between a predominantly proinflammatory or mostly anti‐inflammatory state.^15^ Numerous methods have been described for differentiating THP‐1 monocytes and for polarizing them into either M1 or M2 macrophages.^4^, ^6^, ^16^, ^17^, ^18^ Depending on the desired outcome of a study, different reagents are used to induce differentiation and polarization.^6^, ^17^, ^19^ While differentiation of primary monocytes is induced using granulocyte macrophage colony‐stimulating factor (GM‐CSF) or macrophage colony‐stimulating factor (M‐CSF), THP‐1 cells require protein kinase C activators, such as phorbol 12‐myristate 13‐acetate (PMA) or byrostatin for differentiation into macrophages.^20^ Either interleukin 4 (IL‐4) or a combination of IL‐4 and interleukin 13 (IL‐13), tumor growth factor‐β1 (TGF‐β1), or interleukin 10 (IL‐10) can be used for polarization of THP‐1 monocyte‐derived macrophages (MDMs).^7^, ^18^ Comparison of studies using a variety of methods, however, is problematic due to the lack of standardized and established procedures that use well‐defined macrophage subtypes.

Macrophages are characterized by their high plasticity, changing their cellular metabolism to switch between M1‐ and M2‐like phenotypes.^21^ This dichotomous model cannot fully describe their characteristics, as macrophages, specifically tumor‐associated macrophages (TAM), do not exclusively express either M1‐ or M2‐like markers. Macrophage characterization based on this paradigm is, however, widely used and the fundamental basis for further functional analyses.^5^, ^22^ Proinflammatory M1‐like markers, such as CD80, TNFα, CXCL10, tend to be associated with an antitumor immune response, while macrophages expressing other proinflammatory cytokines (IL‐1β, IL‐6) can mediate tumor cell migration and cancer progression.^23^, ^24^ Predominantly anti‐inflammatory markers, such as CD206, IL‐10, IL‐8, CCL8, and CCL22 are known to be associated with advanced tumor stage and poor survival.^25^

Furthermore, TAM express tumor‐promoting mediators that are involved in tumor neovascularization, such as matrix metalloproteinases (MMP) and ADAM metallopeptidase with thrombospondin type 1 motif 1 (ADAMTS1).^26^, ^27^ The transcription factor activator protein 1 (AP‐1) is closely related to cell proliferation differentiation and apoptosis and mediates proinflammatory cytokine expression in macrophages.^28^ Alpha/beta‐hydrolase domain containing 5 (ABHD5), a key enzyme in lipolysis regulating tumor biology^29^ and toll‐like receptor 4 (TLR4), induced by fatty acids and mediating nuclear factor‐κB (NFκB) pathways, both also play an important role in TAM‐mediated tumor progression.^30^ Another cancer‐related gene involved in lipid metabolism and macrophage activation is monoacylglycerol lipase (MGLL) contributing to lipid accumulation in macrophages and regulating tumor progression.^30^

In the tumor microenvironment, TAMs predominately have anti‐inflammatory M2‐like attributes that are particularly associated with tumor progression and poor overall patient survival.^31^, ^32^, ^33^ In colon cancer, the M2a subtype with a STAT6‐stimulated pathway plays a particular role.^34^, ^35^ In order to investigate the mechanisms mediated by anti‐inflammatory TAMs in a cell culture model, a distinct TAM‐like M2 macrophage subtype is required. Therefore, in vitro inflammatory stimuli must be overcome, including the mechanical stress to the cells as a consequence of media changes, and/or cell treatment with proinflammatory compounds, such as PMA to induce monocyte differentiation.^36^, ^37^

The aim of this study was characterize THP‐1 MDMs after differentiation and polarization into a distinct M2a subtype. The protocol for creating these M2‐like macrophages has been previously described.^38^ Herein, cytokine and tumor‐associated marker gene expression, protein secretion, and cell surface marker expression of cells within this model were investigated and validated as a distinct TAM‐like M2 phenotype.

## 2 MATERIALS AND METHODS

Macrophages were created based on our 14‐day cell line protocol^38^ and characterized according to their marker profile using quantitative real‐time PCR (qRT‐PCR), enzyme‐linked immunosorbent assay (ELISA), flow cytometry, and RNA‐sequencing analysis.

Proinflammatory markers for the characterization of cell types included cluster of differentiation 80 (CD80), tumor necrosis factor alpha (TNFα), interleukin 1β (IL‐1β), interleukin 6 (IL‐6), C‐X‐C motif chemokine ligand 10 (CXCL10). Anti‐inflammatory markers included cluster of differentiation 206 (CD206), IL‐10, IL‐8/CXCL8, C‐C motif chemokine ligand (CCL)18, and CCL22. Specific tumor‐associated markers were MMP2, MMP7, MMP9, MMP12, ABHD5, ADAMTS1, AP‐1, MGLL, and TLR4.

### 2.1 Cell culture and treatment

**FIGURE 1:**
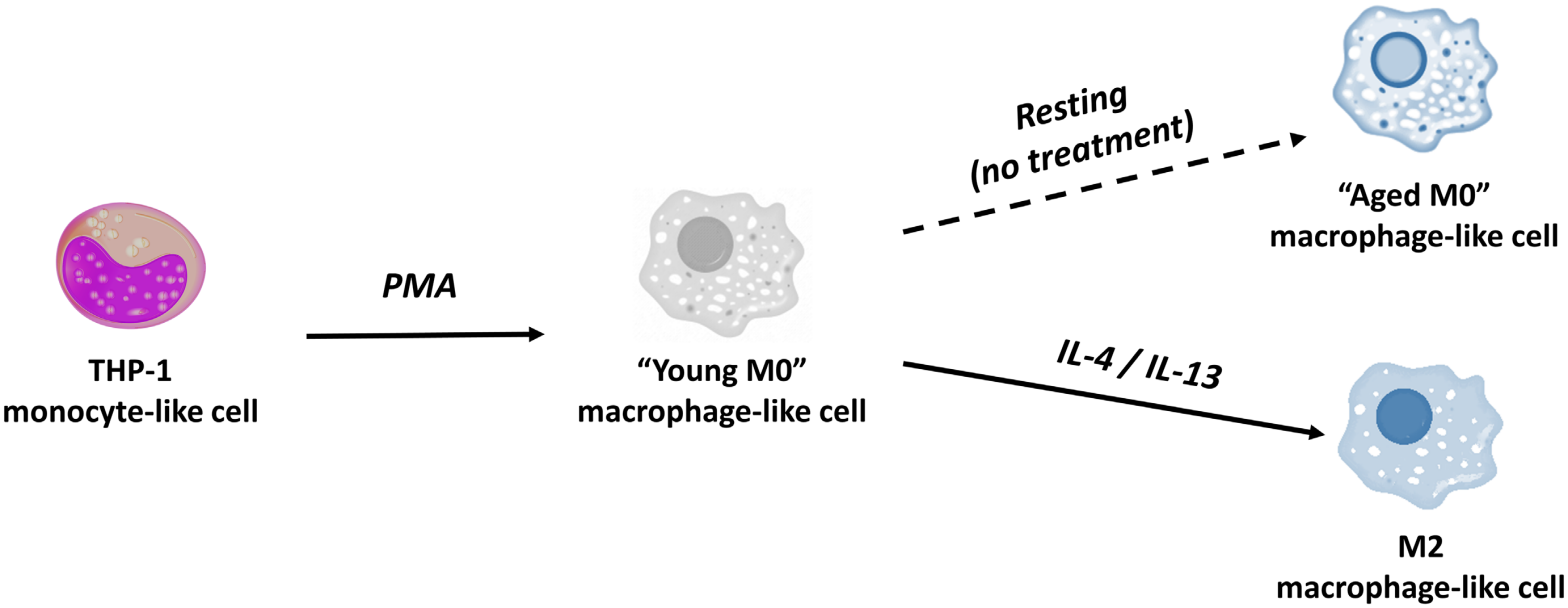
Model for differentiation and polarization of THP‐1 monocytes into a distinct M2‐like macrophage phenotype. This model was based on our 14‐day cell line model for M2‐like macrophages.^38^ THP‐1 monocytes are incubated in growth medium with PMA (“young M0” macrophage). Differentiated macrophages are then polarized using IL‐4 and IL‐13 (M2‐like macrophage). Alternatively, young M0 macrophages were incubated in growth medium alone for 5 days (“aged M0” macrophages). PMA, phorbol 12‐myristate‐13‐acetate; IL, interleukin. Figure modified from Scheurlen et al. “macrophage differentiation and polarization into an M2‐like phenotype using a human monocyte‐like THP‐1 leukemia cell line.”^38^

The human leukemia cell line THP‐1 (TIB202, RRID: CVCL_0006) was purchased from the American Type Culture Collection (ATCC, Manassas, USA) and authenticated using short tandem repeat analysis.^14^ Cells were incubated in RPMI‐1640 medium (ATCC, Manassas, USA) supplemented with 10% fetal bovine serum (FBS) (ATCC, Manassas, USA), 10,000 units/mL penicillin, 10 mg/mL streptomycin, 25 μg/mL amphotericin B, and maintained at 37°C with 5% CO_2_. THP‐1 cells were seeded at 3 × 10^5^ cells/mL into 24‐well cell culture plates and transformed into a TAM‐like M2 phenotype within 14 days (Figure 1). Cells were differentiated into “young M0” macrophages using 100 ng/mL PMA (Sigma‐Aldrich) for 72 h. After 48 h of rest in medium without PMA, the cells were polarized into an M2‐like anti‐inflammatory phenotype by treatment with 20 ng/mL IL‐4 (R&D Systems, Minneapolis, MN, USA) and 20 ng/mL IL‐13 (R&D Systems, Minneapolis, MN, USA) for 96 h. In order to compare macrophages that were actively polarized into an M2‐like phenotype with M0 macrophages that rested without polarization treatment, an alternative “aged M0” macrophage type was created by allowing differentiated macrophages to rest in growth medium alone for 5 days after PMA treatment rather than polarizing them with interleukins. The protocol for creating the final M2a macrophage subtype only has been published elsewhere (doi: 10.3791/62652).^38^

### 2.2 Quantitative real‐time PCR

Total RNA was isolated from THP‐1 cells (*n* = 16), “young M0” (*n* = 32), “aged M0” (*n* = 40), and M2 macrophages (*n* = 60) using the RNeasy purification kit (Qiagen, Germantown, MD, USA) and quantified with spectrophotometry (NanoDrop 1000, Thermo Scientific, Waltham, MA, USA). The sample sizes represent individual wells of cells harvested and are the result of the experimental setup (Figure S1 and S2). The high‐capacity cDNA reverse transcription kit (Applied Biosystems, Foster City, CA, USA) was used with 20 ng of total RNA to perform reverse transcription to cDNA according to the manufacturer's protocol. TaqMan PCR was run with TaqMan gene expression assays (CCL18: HS00268113_m1, CCL22: HS01574247_m1, CD80: HS01045161_m1, CD206/MRC‐1: HS00267207_m1, CXCL10: HS00171042_m1, IL‐1β: HS01555410_m1, IL‐6: HS00174131_m1, CXCL8/IL‐8: HS00174103_m1, IL‐10: HS00961622_m1, TNFα: HS00174128_m1, RNA18S5: Hs03928990_g1, MMP2: HS01548727_m1, MMP7: HS01042796_m1, MMP9: HS00957562_m1, MMP12:HS00159178_m1, ABHD5: HS01104373_m1, ADAMTS1: HS00199608_m1, AP‐1: HS00171851_m1, MGLL: HS00996004_m1, TLR4: HS00152939_m1) (Applied Biosystems) and Fast Advanced Master Mix (Applied Biosystems) using StepOne Real‐Time PCR systems (Applied Biosystems). Results for each target gene were normalized to 18S as the housekeeping gene and are given as mean ΔCT values.

### 2.3 Cell supernatant preparation and enzyme‐linked immunosorbent assay

At baseline and after cell differentiation and polarization, respectively, growth medium was changed to RPMI‐1640 without supplements. Cell supernatants were collected and spun down at 1600 rpm for 7 min and separated from remaining cells.

IL‐10 and IL‐8 concentrations were measured in the supernatants of THP‐1 monocytes, “young M0,” “aged M0,” and M2‐like macrophages according to the manufacturer's protocol using human IL‐10 and human IL‐8 ELISA Kits (Invitrogen, California, USA).

### 2.4 Flow cytometry

Macrophages were lifted from 24‐well plates using the cold shock method by incubating cells with ice‐cold PBS/5% FBS and placing them on ice for 45 min. Following this step, cells were mechanically detached using cell scrapers and washed with cold PBS/5% FBS.

THP‐1 cells (*n* = 5), young M0 (*n* = 5), and M2‐like cells (*n* = 5) were investigated. Nonspecific binding of staining antibodies was inhibited by incubation with Fcγ‐receptor block (BD Pharmingen, San Diego, USA) at room temperature for 10 min. Cells were then stained according to the manufacturer's instructions (BD Parmingen, San Diego, USA) with FITC‐conjugated mouse anti‐human CD14 and CD80 antibodies, with PE‐conjugated mouse anti‐human CD11b antibodies and with PE‐Cy5‐conjugated mouse anti‐human CD206 antibodies and their isotype‐matched IgG for 30 min at 4°C. Cells were then fixed with 1% formaldehyde. 20,000 cells were acquired for each measurement.

Four‐color flow cytometric analysis was performed and fluorescence quantitated using a BD FACSCalibur flow cytometer with CellQuest software (BD Biosciences, San Diego, USA). Gating of cells was carried out to exclude cell debris according to forward and side scatter. Cell viability after processing cells for flow cytometry with detachment of macrophages from cell culture plates was determined in a representative set of samples (*n* = 6) after incubating the cells with 7‐aminoactinomycin‐D (7‐AAD) for 5 min.

### 2.5 Statistical analysis

#### 2.5.1 Quantitative real‐time PCR and ELISA analysis

For qRT‐PCR analysis, all delta CT values between cell types were compared using a one‐way analysis of variance (ANOVA) model with a post hoc Benjamini–Hochberg correction to control the false discovery rate at 5%.^39^ ELISA cytokine concentrations were compared using a one‐way ANOVA with a Bonferroni test.^40^ The heat map for qRT‐PCR was created using R software and pirate plots for ELISA results were generated using the software R package “yarrr.”^41^, ^42^

#### 2.5.2 RNA‐sequencing analysis

##### Sample collection

For the cell line analysis, three THP‐1‐derived M2‐like macrophage samples were created according to our protocol and sequenced by the HudsonAlpha Genome Sequencing Center (Huntsville, AL). Two corresponding THP‐1‐derived peripheral blood monocyte samples were downloaded in fastq format from Gene Expression Omnibus (GEO)^43^ (data accessible at NCBI GEO database, Phanstiel et al. 2017, accession GSE96800 [samples GSM2599707 and GSM2599708]). Raw read counts from a third THP‐1 peripheral blood monocyte sample were downloaded from the Dependency Map Portal (DepMap)^44^ with the corresponding ID ACH‐000146.

For the primary tissue analysis, raw single‐cell read counts for human TAMs from colon cancer core tissue were obtained from the Single Cell Atlas accession E‐MTAB‐8410.^45^ E‐MTAB‐8410 samples were gathered from nine patients and covered several tissue types: colon, caecum, ascending colon, sigmoid colon, and rectosigmoid colon. Three samples were collected from each patient: tumor cells, tumor border cells, and adjacent normal cells. Raw single‐cell read counts for human peripheral blood monocytes derived from colon cancer patients were obtained from GEO (data accessible at NCBI GEO database, accession GSE178318).^46^

An overview of the data is shown in Table S1.

##### Cell line RNA‐sequencing analysis

The THP‐1‐derived M2‐like TAM cell lines sequenced by HudsonAlpha and the two THP‐1 peripheral blood monocyte cell lines retrieved from GEO were aligned to reference assembly hg38 using RSEM.^47^ Expected counts were generated with Gencode annotations V34. The expected counts for a third monocyte cell line retrieved from DepMap were previously generated by RSEM using Gencode V34. Raw counts for the three TAM cell lines and the three monocyte cell lines were input to DESeq2^48^ for differential expression analysis which uses relative log expression as its default normalization method.

##### Primary tissue RNA‐sequencing analysis

Raw counts for peripheral blood mononuclear cells (PBMCs) from GSM2599708 were integrated with raw counts for all cells from E‐MTAB‐8410 using Seurat.^49^ Available metadata were also input to Seurat. Cells with greater than 6000 features (possible doublets), fewer than 200 features (poor capture), and greater than 20% mitochondrial reads (possible dead and dying cells) were removed prior to integration. As part of the integration process, scaling and principal component analysis were performed on the individual samples. Integration anchors were identified using RPCA reduction with up to 30 dimensions specified. Scaling, dimension reduction (PCA/UMAP), and clustering at a resolution of 0.5 was performed on the integrated data. The integrated Seurat object was uploaded to BioTuring's BBrowserX^50^ for cell type prediction. Version 3 of BioTuring's machine learning algorithm, which uses 84 million cells as a reference, was selected for cell type prediction. TAMs were identified by considering the annotations provided by Lee et al. in the original study (anti‐inflammatory myeloid cell, SPP1 + B myeloid cell, SPP1 + A myeloid cell, and pro‐inflammatory myeloid cell) along with BioTuring's BBrowserX annotations (macrophage). One set of TAMs includes the 103 cell annotations in agreement across the two methods, which was analyzed and is presented in the results section. A second set includes the 103 TAM annotations from Set 1 and all additional macrophage annotations by Lee et al., which resulted in analyzing 656 TAMs (Data S1). Differential expression between TAMs and PBMC monocytes was performed for each set of TAMs using Seurat's findMarkers function and the Wilcoxon rank sum test.

## 3 RESULTS

### 3.1 Expression of pro‐ and anti‐inflammatory markers increases during development of THP‐1 monocytes into TAM‐like M2 macrophages

**FIGURE 2:**
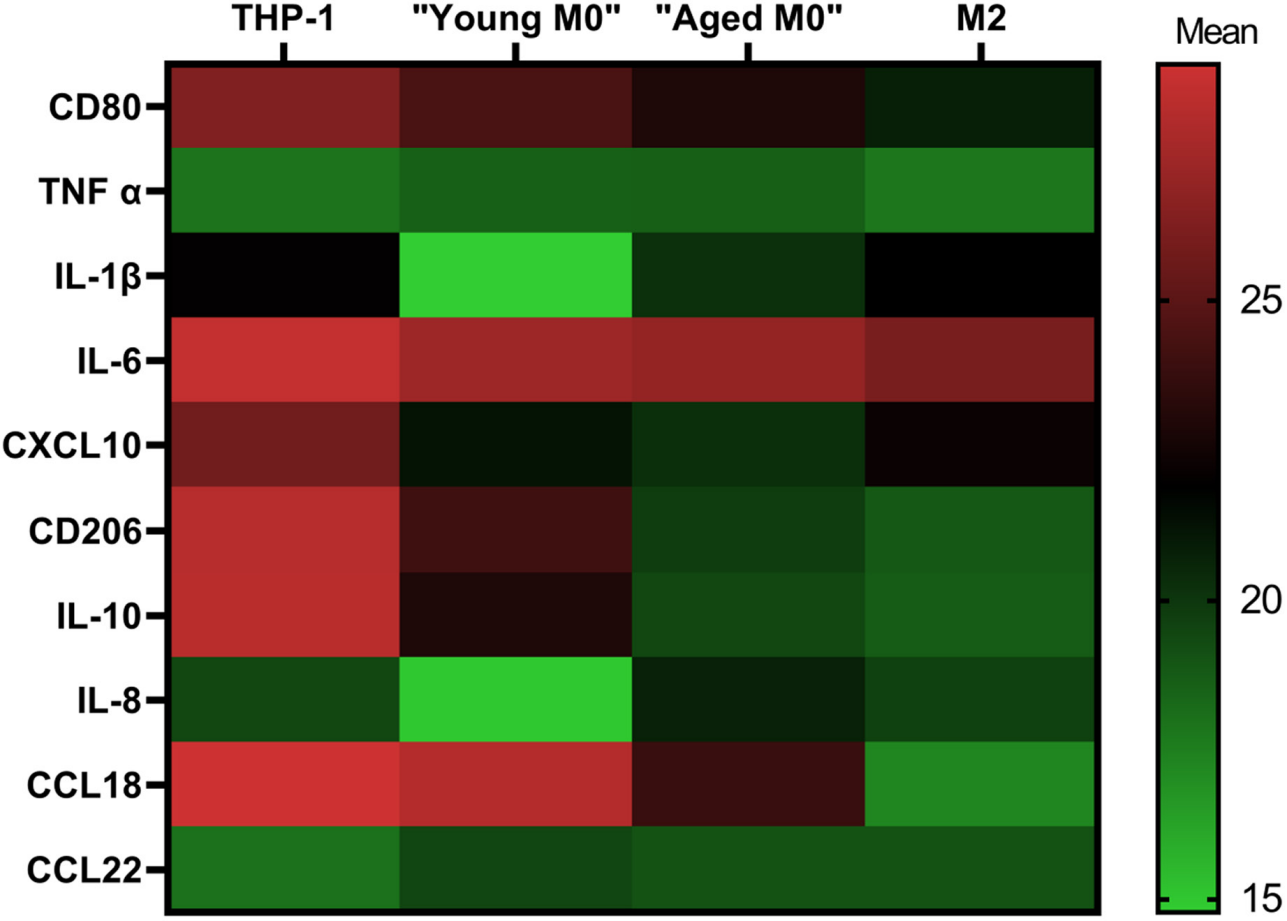
Marker gene expression using qRT‐PCR among cell types. Heat map of marker gene expression (ΔCT values) of pro‐ and anti‐inflammatory markers among all cell types. Lowest gene expression is depicted by red, highest expression by green. CCL, C‐C motif chemokine ligand; CD, cluster of differentiation; CXCL, C‐X‐C motif chemokine ligand; TNFα, tumor necrosis factor α; IL, interleukin.

Pro‐ and anti‐inflammatory marker expression was significantly different among all cell types for CD80, TNFα, IL‐1β, CXCL10, CD206, IL‐10, IL‐8/CXCL8, CCL18, and CCL22; each with *p* < 0.001, and for IL‐6 *p* = 0.033 (Tables 1 and 2). Expression patterns (mean ΔCT values) of all markers for the respective cell types are shown in Figure 2 and *p*‐values of pairwise comparisons are given in Table S2.

**TABLE 1:** Proinflammatory markers expression (ΔCT values) among cell types.

Marker	THP‐1 (ΔCT), *N* = 16	Young M0 (ΔCT), *N* = 32	Aged M0 (ΔCT), *N* = 40	M2 (ΔCT), *N* = 60	*p*‐value	Significant pairwise differences
CD80	26.27 ± 1.55	24.36 ± 3.37	22.87 ± 0.58	20.79 ± 0.54	<0.001***	All
TNFα	17.88 ± 1.47	18.53 ± 0.91	18.57 ± 0.45	17.97 ± 0.73	<0.001***	THP‐1—young M0; THP‐1—Aged M0; Young M0—M2; Aged M0—M2
IL‐1β	21.96 ± 0.51	14.78 ± 3.54	20.18 ± 0.72	21.84 ± 0.55	<0.001***	THP‐1—Young M0; THP‐1—Aged M0; Young M0—Aged M0; Young M0—M2; Aged M0—M2
IL‐6	28.59 ± 1.22	27.26 ± 1.74	26.92 ± 6.41	26.04 ± 1.53	0.033*	THP‐1—M2
CXCL10	25.75 ± 1.24	21.20 ± 0.89	20.21 ± 1.52	22.34 ± 0.76	<0.001***	All

*Note*: Marker expression was investigated using quantitative real‐time PCR. Results are shown as mean ΔCT values and standard deviations (SD) for all cell types. Significant results of pairwise comparisons between cell types are listed.

Abbreviations: CD, cluster of differentiation; CXCL, C‐X‐c motif chemokine ligand; IL, interleukin; TNFα, tumor necrosis factor α.

*p* < 0.05 indicates a significant pairwise comparison. *p < 0.05, **p ≤ 0.01, ***p ≤ 0.001.

**TABLE 2:** Anti‐inflammatory marker expression (ΔCT values) among cell types.

Marker	THP‐1 (ΔCT), *N* = 16	Young M0 (ΔCT), *N* = 32	Aged M0 (ΔCT), *N* = 40	M2 (ΔCT), *N* = 60	*p*‐value	Significant pairwise differences
CD206	28.15 ± 1.14	23.99 ± 0.71	19.73 ± 0.50	18.80 ± 0.47	<0.001***	All
IL‐10	28.22 ± 1.55	22.88 ± 0.67	19.38 ± 0.44	18.70 ± 0.47	<0.001***	All
IL‐8	19.41 ± 1.39	14.93 ± 2.21	20.69 ± 0.82	19.45 ± 0.43	<0.001***	THP‐1—Young M0; THP‐1—Aged M0; Young M0—Aged M0; Young M0—M2; Aged M0—M2
CCL18	28.95 ± 0.91	28.06 ± 0.95	23.80 ± 0.42	17.23 ± 0.34	<0.001***	All
CCL22	17.96 ± 0.55	19.45 ± 1.10	19.03 ± 0.46	19.03 ± 0.40	<0.001***	THP‐1—Young M0; THP‐1—Aged M0; THP‐1—M2; Young M0—Aged M0; Young M0—M2

*Note*: Marker expression was investigated using quantitative real‐time PCR. Results are shown as mean ΔCT values and standard deviations (SD) for all cell types. Significant results of pairwise comparisons between cell types are listed.

Abbreviations: CCL, C‐C motif chemokine ligand; CD, cluster of differentiation; CXCL, C‐X‐C motif chemokine ligand; IL, interleukin.

*p* < 0.05 indicates a significant pairwise comparison. *p < 0.05, **p ≤ 0.01, ***p ≤ 0.001.

#### 3.1.1 M1‐associated marker expression increases, but not interleukin‐1β expression

Overall *p*‐values with means and SDs of PCR ΔCT values for all cell types are shown in Table 1.

Pairwise comparisons between groups demonstrated a significant difference of M1‐associated CD80 between cell types due to upregulation of CD80 in “young M0,” “aged M0,” and M2 macrophages compared to THP‐1 monocytes (Figure 2, Table 1).

The pro‐inflammatory marker TNFα was significantly downregulated in “young M0” and “old M0” compared to M2 macrophages and compared to THP‐1 monocytes. The difference between M2‐macrophages and THP‐1 cells at baseline was not statistically significant (Figure 2, Table 1).

The M1‐associated marker IL‐1β was significantly upregulated in “young M0” and “aged M0” macrophages compared to THP‐1 cells as well as compared to M2 subtype macrophages. “Young M0” macrophages showed the highest level of IL‐1β expression (Figure 2, Table 1).

IL‐6 was significantly upregulated in “young M0,” “aged M0,” and M2 macrophages compared to THP‐1 cells (Figure 2, Table 1).

CXCL10 showed a significantly higher expression in “young M0,” “aged M0,” and M2 macrophages compared to THP‐1 monocytes. The highest level of CXCL10 expression was demonstrated in both M0 cell groups (Figure 2, Table 1).

#### 3.1.2 M2‐associated marker expression increases, but not interleukin‐8 expression

Means, SDs, and overall *p*‐values of PCR ΔCT values for all cell types are shown in Table 2.

CD206 was most highly expressed in M2 macrophages, with “young M0,” “aged M0,” and M2 cells showing significant upregulation compared to THP‐1 monocytes (Figure 2, Table 2).

The M2‐associated marker IL‐10 showed the same expression pattern as CD206, with the highest expression in M2 macrophages. “Aged M0” macrophages showed significantly higher CD206 expression compared to “young M0” macrophages or THP‐1 cells, (Figure 2, Table 2).

IL‐8/CXCL8 was significantly upregulated in “young M0” macrophages compared to “aged M0,” M2, and THP‐1 cells.

The highest M2‐associated CCL18 expression was seen in M2 macrophages with significant upregulation compared to all other cell types. “Aged M0” cells showed a significantly higher expression compared to “young M0” and THP‐1 cells (Figure 2, Table 2).

CCL22 was significantly downregulated in all cell types compared to THP‐1 cells. (Figure 2, Table 2).

### 3.2 Expression of tumor‐associated markers increases in transition from THP‐1 monocyte to TAM‐like M2 macrophage

Tumor‐associated marker expression was significantly different among all cell types for MMP2, MMP7, MMP9, MMP12, ABHD5, ADAMTS1, AP‐1, and MGLL each with *p* < 0.001 (Table 3). TLR4 expression showed no significant difference. The corresponding p‐values of pairwise comparisons are given in Table S2.

**TABLE 3:** Tumor‐associated marker expression (ΔCT values) among cell types.

Marker	THP‐1 (ΔCT), *N* = 16	Young M0 (ΔCT), *N* = 32	Aged M0 (ΔCT), *N* = 40	M2 (ΔCT), *N* = 60	*p*‐value	Significant pairwise differences
MMP2	19.59 ± 0.27	19.13 ± 1.10	16.72 ± 0.36	17.87 ± 0.34	<0.001***	All
MMP7	28.33 ± 3.21	18.29 ± 0.68	25.30 ± 0.92	22.92 ± 0.61	<0.001***	All
MMP9	16.55 ± 0.63	10.02 ± 0.48	11.15 ± 0.38	10.59 ± 0.42	<0.001***	All
MMP12	27.31 ± 1.80	19.62 ± 2.41	22.80 ± 0.66	19.04 ± 0.64	<0.001***	THP‐1—Young M0; THP‐1—Aged M0; THP‐1—M2; Young M0—Aged M0; Aged M0—M2
ADAMTS1	19.39 ± 0.32	20.40 ± 1.54	20.29 ± 0.71	21.61 ± 1.13	<0.001***	THP‐1—Young M0; THP‐1—Aged M0; THP‐1—M2; Young M0—M2; Aged M0—M2
ABHD5	18.47 ± 0.32	18.29 ± 0.63	18.78 ± 1.03	19.68 ± 1.54	<0.001***	THP‐1—M2; Young M0—M2; Aged M0—M2
MGLL	21.96 ± 0.46	16.74 ± 1.51	16.94 ± 0.46	18.25 ± 0.37	<0.001***	THP‐1—Young M0; THP‐1—Aged M0; THP‐1—M2; Young M0—M2; Aged M0—M2
TLR4	20.24 ± 0.25	18.27 ± 0.71	19.18 ± 3.43	18.77 ± 2.50	0.056	
AP‐1	25.80 ± 1.20	25.05 ± 2.90	27.00 ± 0.89	26.25 ± 1.48	<0.001***	THP‐1—Young M0; Young M0—M2; Aged M0—M2

*Note*: Marker expression was investigated using quantitative real‐time PCR. Results are shown as mean ΔCT values and standard deviations (SD) for all cell types. Significant results of pairwise comparisons between cell types are listed.

Abbreviations: ABHD5, alpha/beta‐hydrolase domain containing 5; ADAMTS1, ADAM metallopeptidase with thrombospondin type 1 motif 1; AP‐1, activator protein 1; MGLL, monoglyceride lipase; MMP, matrix metalloproteinase; TLR4, toll‐like receptor 4.

*p* < 0.05 indicates a significant pairwise comparison. *p < 0.05, **p ≤ 0.01, ***p ≤ 0.001.

All investigated tumor‐promoting MMPs were significantly increased in M2‐like macrophages, with MMP2 showing the lowest upregulation in M2‐like macrophages compared to THP‐1 monocytes (Table 3). The metallopeptidase ADAMTS1 was significantly downregulated in M2‐like, “young M0” and “aged M0” macrophages compared to THP‐1 cells (Table 3). ABHD5, a lipolytic factor either potentiating tumor growth or functioning as a tumor suppressor in certain types of cancer, was significantly downregulated in M2‐like macrophages compared to all other cell types (Table 3). Upregulation of MGLL, a key enzyme in lipid metabolism that also has both tumor promoting and suppressing effects, was most evident in both M0 phenotypes compared to THP‐1 monocytes, but also significant in M2‐like macrophages (Table 3).

The immune response receptor TLR4 showed no significantly different expression among cell types. The transcription factor AP‐1 exerting a dual role among different types of cancers, showed a slight upregulation in “young M0” macrophages compared to THP‐1 cells, but no difference was found in M2‐like macrophages versus THP‐1 (Table 3).

#### 3.2.1 Expression of M2a‐specific subtype markers in cell line‐derived TAM‐like M2 macrophages

Expression of M2a‐specific subtype markers was significantly upregulated in cell line‐derived TAM‐like M2 macrophages compared to THP‐1 monocytes (Table S3).

CHI3L1, PTPRC, and ITGAM, three key genes in colon cancer proliferation and metastasis, demonstrated the highest baseline expression and significant changes in gene expression. CHI3L1 was downregulated in TAM‐like M2 macrophages (*p* < 0.001), while PTPRC and ITGAM were upregulated (*p* < 0.001). STAB1, CLEC4A, and IGF1 showed the highest fold change in THP‐1 monocytes at baseline (11.7, *p* < 0.001; 10.5, *p* < 0.001; and 7.9, *p* < 0.001).

CHI3L2, CCL1, CCL17, ARG1, and F8A3 had a low baseline expression and no significant increase.

### 3.3 TAM‐like M2 macrophages show increased protein expression of interleukin‐10, but not interleukin‐8 compared to THP‐1 monocytes

**FIGURE 3:**
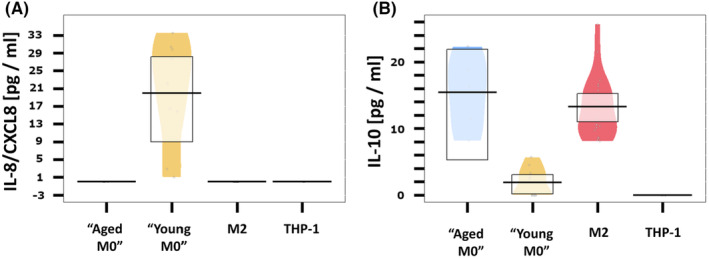
Pirate plots of protein expression of anti‐inflammatory markers among cell types (A) interleukin 8 (IL‐8), (B) interleukin 10 (IL‐10). IL, interleukin.

Protein expression of the anti‐inflammatory cytokine IL‐8/CXCL8 was significantly different among cell types (*p* < 0.001; Figure 3A). IL‐8/CXCL8 protein expression did not significantly differ between THP‐1 cells, “aged M0” cells and M2 macrophages. “Young M0” cells showed a variable, but significantly increased IL‐8/CXCL8 protein expression (19989.56 [95% CI 12930.32, 27048.80] pg/mL) compared to the other cell types.

Protein expression of the M2‐like cytokine IL‐10 differed significantly among cell types (*p* < 0.001) (Figure 3B). IL‐10 was not detectable in THP‐1 cells. There was no significant IL‐10 protein expression in “young M0” macrophages. IL‐10 protein was significantly expressed in “aged M0” (15.43 [95% CI 9.82, 20.98] pg/mL) and in M2 macrophages (13.29 [95% CI 8.75, 17.82] pg/mL), with no significant difference between these two cell types.

### 3.4 Cell line‐derived M2 macrophages express anti‐inflammatory cell surface markers

Cell viability of differentiated macrophages after incubating cells with 7‐AAD was >95% throughout all samples.

The myeloid marker CD14 was expressed in 2.06% ± 0.46% of THP‐1 cells. “Young M0” macrophages showed low CD14 expression (3.87% ± 1.81% of cells). There was, however, a distinct upregulation of CD14, which was demonstrated in 69.18% ± 3.48% of M2 macrophages.

THP‐1 monocytes exhibited low CD11b expression (4.39% ± 1.36% of cells). A significant upregulation of CD11b could be demonstrated in M2 macrophages (76.37% ± 4.68%), with a lower expression in “young M0” macrophages (11.47% ± 2.65%).

The M1‐like marker CD80 was not markedly expressed in any cell type (0.11% ± 0.07% of THP‐1 cells; 0.36% ± 0.17% of M0 cells and 0.05% ± 0.01% of M2 macrophages).

Expression of the M2‐like marker CD206 was low among THP‐1 cells (0.22% ± 0.07%) and “young M0” macrophages (0.98% ± 0.64%), with a significant upregulation in M2 macrophages (58.08% ± 2.74%).

### 3.5 The RNA‐sequencing profile of cell line‐derived TAM‐like M2 macrophages resembles the profile of primary human colon cancer TAMs

#### 3.5.1 Inflammatory and tumor‐associated genes change significantly in TAM‐like M2 macrophages compared to THP‐1 monocytes

RNA‐Seq data were used to investigate the expression level of a respective gene in the baseline cell line monocyte and to compare changes in cell line‐derived M2‐like macrophages (Table 4).

**TABLE 4:** RNA‐Seq marker expression in cell line THP‐1 monocytes (*N* = 3) versus TAM‐like M2 macrophages (*N* = 3).

Marker	Mean baseline expression	Mean log_2_ FC	Adjusted *p*‐value
CD80	22.7	3.5	0.026*
CD86	1400.2	6.9	<0.001***
TNFα	205.8	−0.9	0.128
IL‐1β	25.1	−0.5	0.528
IL‐6	1.4	−0.6	n/a
CXCL10	8.9	2.8	0.046*
CD206	1690.7	12.0	<0.001***
IL‐10	68.3	8.3	<0.001***
IL‐8	46.9	−0.2	0.792
CCL18	1081.1	12.9	<0.001***
CCL22	85.3	−0.1	0.894
MMP2	616.2	1.8	0.028*
MMP7	9.5	3.3	0.032*
MMP9	12960.5	11.8	<0.001***
MMP12	53.0	7.9	<0.001***
ADAMTS1	234.1	−3.3	<0.001***
ABHD5	298.4	−0.2	0.631
MGLL	210.8	3.2	<0.001***
TLR4	1690.7	3.4	<0.001***
AP‐1	7.2	−0.7	0.579

Abbreviations: ABHD5, alpha/beta‐hydrolase domain containing 5; ADAMTS1, ADAM metallopeptidase with thrombospondin type 1 motif 1; AP‐1, activator protein 1; CCL, C‐C motif chemokine ligand; CD, cluster of differentiation; CXCL, C‐X‐C motif chemokine ligand; IL, interleukin; MGLL, monoglyceride lipase; MMP, matrix metalloproteinase; TLR4, toll‐like receptor 4; TNFα, tumor necrosis factor α.

*p* < 0.05 indicates a significant pairwise comparison. *p < 0.05, **p ≤ 0.01, ***p ≤ 0.001.

Baseline expression was exceptionally high for CD86, CD206, CCL18, MMP9, and TLR4. These markers were significantly upregulated in THP‐1‐derived M2‐like macrophages. Proinflammatory CD80, CXCL10, anti‐inflammatory IL‐10, and the tumor‐associated markers MMP2, MMP7, MMP12, and MGLL showed lower baseline expression and were upregulated in macrophages as well. ADAMTS1 was the only downregulated marker.

Single‐cell RNA‐Seq data were analyzed to demonstrate the proportion of cells expressing the respective genes in primary PBMCs compared to primary human colon cancer tissue macrophages with a mean log_2_ fold change (Table 5).

**TABLE 5:** scRNA‐Seq marker expression in peripheral blood monocytes (*N* = 2099) versus primary human colon cancer tissue TAMs (*N* = 103).

Marker	Mean log_2_ FC	Percentage of monocytes	Percentage of macrophages	*p*‐value
CD80	0.1	0.2	6.8	<0.001***
CD86	0.3	30.4	64.1	0.003**
TNFα	−1.0	24.7	1.9	0.004**
IL‐1β	−3.2	74.7	17.5	<0.001***
IL‐6	0	1.9	4.9	1
CXCL10	−0.2	2.7	2.9	1
CD206	1.5	0.4	60.2	<0.001***
IL‐10	0.1	1.3	6.8	0.706
IL‐8	−1.2	84.1	62.1	<0.001***
CCL18	2.3	0.1	50.5	<0.001***
CCL22	0	0	1.9	<0.001***
MMP2	0.3	0	19.3	<0.001***
MMP7	0.7	0	10.7	<0.001***
MMP9	1.0	0.6	24.3	<0.001***
MMP12	2.2	0	24.3	<0.001***
ADAMTS1	0	0	0	1
ABHD5	−0.1	19.2	29.1	1
MGLL	0.4	0.9	23.3	<0.001***
TLR4	0.1	13.6	39.8	<0.001***
AP‐1	−1.9	87.4	52.4	<0.001***

Abbreviations: ABHD5, alpha/beta‐hydrolase domain containing 5; ADAMTS1, ADAM metallopeptidase with thrombospondin type 1 motif 1; AP‐1, activator protein 1; CCL, C‐C motif chemokine ligand; CD, cluster of differentiation; CXCL, C‐X‐C motif chemokine ligand; IL, interleukin; MGLL, monoglyceride lipase; MMP, matrix metalloproteinase; TLR4, toll‐like receptor 4; TNFα, tumor necrosis factor α.

*p* < 0.05 indicates a significant pairwise comparison. *p < 0.05, **p ≤ 0.01, ***p ≤ 0.001.

A high proportion of primary peripheral blood monocytes (>20%) expressed CD86, TNFα, IL‐1β, IL‐8, and AP‐1. The proportion of TAMs expressing these genes was decreased and showed significantly lower expression, except for CD86. Proinflammatory CD80, anti‐inflammatory CD206, CCL18, and CCL22, and the tumor‐associated markers including MMPs, MGLL, and TLR4 were expressed by a low proportion of monocytes (<1%), but significantly upregulated in TAM. ADAMTS1 was neither expressed in monocytes nor TAM.

**FIGURE 4:**
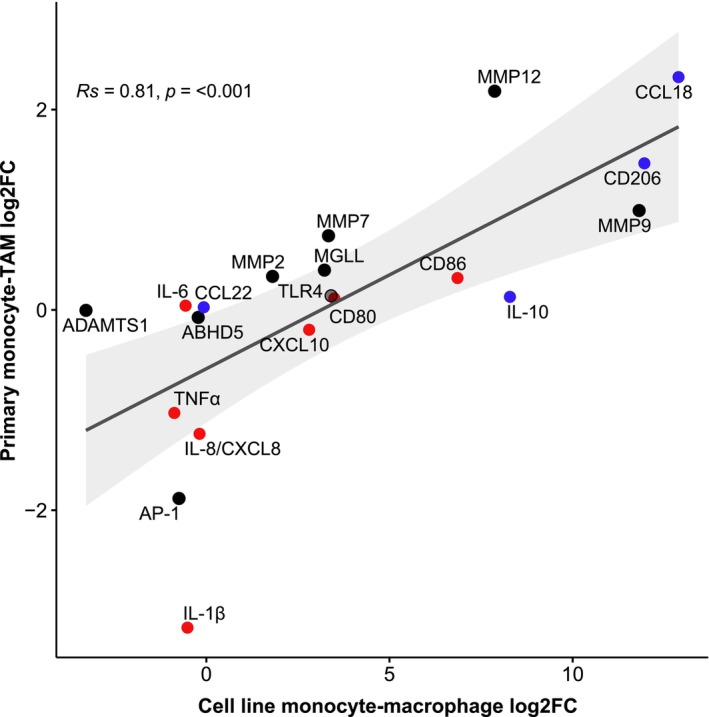
Correlation of changes in gene expression between primary human peripheral blood monocytes (*N* = 2099) and primary human colon cancer tissue TAMs (*N* = 103) and cell line‐derived THP‐1 monocytes (*N* = 3) and cell line‐derived TAM‐like M2 macrophages (*N* = 3). Primary cell data represents single‐cell RNA‐sequencing data, while cell line‐derived results are based on bulk RNA‐sequencing data. Red: proinflammatory gene. Blue: anti‐inflammatory gene. Black: tumor‐associated gene. ABHD5, alpha/beta‐hydrolase domain containing 5; ADAMTS1, ADAM metallopeptidase with thrombospondin type 1 motif 1; AP‐1, activator protein 1; CCL, C‐C motif chemokine ligand; CD, cluster of differentiation; CXCL, C‐X‐C motif chemokine ligand; IL, interleukin; MGLL, monoglyceride lipase; MMP, matrix metalloproteinase; TLR4, toll‐like receptor 4; TNFα, tumor necrosis factor α.

A Spearman correlation was used to investigate the association of log2 fold changes for genes between the cell line‐derived TAM‐like M2 macrophages and primary human TAM. Compared to primary cells from colon cancer patients, cell line‐derived monocyte–macrophage development showed a similar change in cell gene expression profiles (Figure 4). While more profound changes in gene expression could be observed with the cell line as expected, primary cell changes correlated significantly (*R*_s_ = 0.81, *p* < 0.001). Taken together, these data indicate that the gene expression of cell line‐derived TAM‐like M2 macrophages resembles that of primary human colon cancer TAMs.

An additional analysis with the subset of 656 primary colon cancer TAMs labeled by Liu et al. compared to primary human peripheral blood monocytes (*N* = 2099) showed similar confirmatory results (Table S4, Figure S3).

## 4 DISCUSSION

Macrophages mediate central mechanisms in wound healing, fibrosis, and cancer growth. Specifically, the proportion of anti‐inflammatory M2 macrophages is associated with advanced tumor stage and decreased overall survival in certain types of cancers.^32^, ^51^, ^52^ Currently, there is no distinct M2 macrophage subtype based on an established reproducible protocol that makes studies investigating functional analyses in cancer research comparable. Various techniques for differentiating and polarizing macrophages are available, but experimental models are rarely well‐characterized and lack reproducibility.^4^, ^6^, ^16^, ^17^, ^18^ Cell line‐derived macrophage models are based on immortalized cells derived from only a single patient and increase reproducibility by showing more consistent and enhanced cellular responses compared to primary cells.^53^ While models using primary cells have been described to resemble a more complex, and therefore in vivo macrophage response, data obtained using such cells often vary and are difficult to reproduce. Data reproducibility is a key component of funding mandates.^54^ The use of primary human cells is associated with various other disadvantages, such as impaired cell growth, limited replicative capacity, and cell senescence, varying cellular responses due to donor variability, potential ethical concerns, and high cost.^55^, ^56^ On the other hand, cell lines are genetically manipulated, which may alter their responsiveness to stimuli.^53^ Cross‐contamination, misidentification, and mycoplasma contamination of cell lines can alter cellular behavior.^57^, ^58^ Therefore, cell lines should not be used exclusively to investigate functional cellular responses. After identifying a cellular mechanism within cell lines, experiments utilizing primary cells may help in contributing to confirm these mechanisms as a basis for planning future in vivo models.

We have provided a protocol of a cell line model for differentiating and polarizing THP‐1 monocytes within 14 days and herein characterize the distinct TAM‐like M2a macrophage subtype that is thereby created. This provides the basis for investigating mechanisms mediated by anti‐inflammatory macrophages in vitro.^38^ Macrophages can be divided into several subsets from M2a‐d.^59^ These subsets have been investigated in association with the development of different cancers, and their gene expression profiles are overlapping. Macrophage subsets can only be identified by focusing on specific key genes. In our distinct TAM‐like macrophage subtype, an M2a profile could be identified. These macrophages show high STAT6‐related gene expression, induced by IL4 and IL13 exposure,^34^ which is a central mechanism in colon cancer development and progression.^34^, ^35^ Key genes of M2a macrophages play a particular role in colon cancers. ITGAM or CD11b, a marker of macrophage activation, has been shown to be upregulated in colon cancer.^60^ STAB1 is a scavenger receptor on anti‐inflammatory macrophages and was demonstrated to facilitate lymphatic metastasis. Its upregulation is associated with shorter disease‐specific survival.^61^ CCL17 is involved in colon cancer cell migration as well but has not shown to be significantly upregulated in our TAM‐like macrophages.^62^

CLEC4A and TREM2 were significantly upregulated in the created cell line‐derived TAM‐like macrophages described herein. These tumor suppressor genes are linked to colon cancer cell proliferation and development.^63^, ^64^ CLEC4A has been demonstrated to have an impact on colon inflammation and the onset of colitis in particular.^64^ Therefore, our TAM‐like macrophage subtype represents an attractive model to study the underlying inflammatory mechanisms impacting tumor development within functional experiments.

One of the limitations of this study is the use of a human cell line model rather than primary cells as a basis to investigate human macrophage mechanisms in vivo. In contrast, primary human macrophages are characterized by great variation in marker expression patterns and have different functional roles according to their source of origin.^65^ Previous data showed that PMA‐treated THP‐1 macrophages did not completely mimic the complexity of primary MDM activation.^66^ The use of a cell line, however, can provide a reproducible and standardized model to recreate functional responses in vitro. Furthermore, characteristics of THP‐1‐derived cells used for mimicking macrophage mechanisms in vivo can be altered to achieve similar marker profiles to primary macrophages. Another limitation is the complexity of myeloid cell characterization due to cell plasticity and the wide variety of functional abilities of these cells. This leads to limited characterization of M2 macrophages created by this protocol. The cell treatment scheme of this study, however, demonstrates the reproducible creation of a distinct M2‐like phenotype of macrophages, that can be further characterized depending on the intended experimental setting.^38^ Cytokine mRNA and protein expression and flow cytometry used in this study demonstrated a clear anti‐inflammatory shift of differentiated macrophages and differences between resting cells versus cells receiving polarization treatment. Further analyses concerning pro‐ and anti‐inflammatory cytokine expression, protein production, and cell surface marker expression as well as functional analyses of these M2‐like macrophages should follow on the basis of the underlying treatment protocol.

The differentiation of THP‐1 monocytes is widely performed by incubating cells with the proinflammatory compound PMA.^17^, ^36^, ^37^, ^67^ This method of differentiation is especially suitable for mimicking primary human MDM, showing similar characteristics concerning cell morphology, macrophage surface markers, and cytokine profiles.^68^ Pro‐inflammatory responses of the macrophages that are created by this approach must decrease in order to obtain non‐polarized M0 macrophages and to assure an adequate M2‐like polarization process. For this reason, cells are reported to rest in medium only after PMA treatment for various periods of time.^6^, ^16^, ^67^ This model is based on a 5‐day rest period after PMA incubation, creating macrophages that mimic primary human MDM in terms of cytoplasmatic volume and cell surface adherence.^67^, ^69^

The cytokine profile of young M0 macrophages that are described in this study demonstrated significantly increased expression of the proinflammatory markers CD80, IL‐1β, IL‐6, and CXCL10 compared to THP‐1 monocytes, while no difference in TNFα expression was shown. Primary colon cancer cells showed downregulation of TNFα. Even though the term “M0 macrophages” defines differentiated macrophages in a resting state in vitro, characteristics of cellular activation (M1‐like state) are usually identified in the real‐world setting.^70^ Therefore, the standardized model presented herein clearly describes cytokine expression as a baseline threshold for future experiments. The anti‐inflammatory marker IL‐8, however, is most highly expressed in young M0 macrophages and significantly upregulated in THP‐1‐derived M2‐like macrophages compared to all the other cell stages. This was confirmed by protein expression analysis, showing high variability of IL‐8/CXCL8 concentrations among “young M0” macrophages. RNA‐Seq data of colon cancer TAMs showed IL‐8 expression in a high proportion of cells with downregulation compared to PBMCs. Variation in expression levels of IL‐8/CXCL8 is a previously described characteristic of this cytokine in several different cell types, such as pulmonary epithelial cells or cervical cancer cells.^71^ THP‐1‐derived macrophages have also been reported to show highly variable IL‐8/CXCL8 protein expression after treatment with proinflammatory stimuli.^69^, ^71^ This observation is partially understood to result from the functional role of IL‐8/CXCL8, which involves synchronization of several regulatory pathways, including NFκ‐B, c‐Jun N‐terminal kinase (JNK), and p38 mitogen‐activated protein kinase (p38 MAPK).^69^

The M2‐like macrophages created by this model demonstrate a significant upregulation of the anti‐inflammatory markers CD206, IL‐10, and CCL18 compared to both “young M0” and “aged M0” macrophages, with CCL18 upregulation showing the greatest difference in M2‐like cells as compared to either type of M0 cells. These results could be confirmed by primary colon cancer TAM scRNA‐Seq data. This demonstrates that the cell line model of this study is mimicking primary human MDM, as CCL18 is also abundantly produced by these cells following M2‐like polarization.^72^ CCL18 induces macrophage maturation into an M2‐like phenotype itself and is known to be produced by TAMs in several different types of cancers.^73^, ^74^, ^75^ Both groups of M0 macrophages did not differ from M2‐like cells with respect to CCL22 expression. Production of IL‐10 in M2‐like macrophages was confirmed by a significantly increased IL‐10 protein expression in these cells compared to that observed in THP‐1 monocytes and young M0 macrophages. IL‐10 production in “aged M0” macrophages did not differ from M2 macrophages on the protein level, however, the variability in IL‐10 production was high among “aged M0” cells. This was confirmed by our scRNA‐Seq data analysis of primary TAMs. IL‐10 is reported to be upregulated in polarized primary human MDM as well, mostly as part of an autocrine mechanism using the signal transducer and activator of transcription 3 (STAT3) pathway.^8^

Flow cytometry analysis for cell surface expression markers revealed that the distinct M2‐like subtype that is created by this protocol not only shows high expression of the anti‐inflammatory marker CD206, but also of both myeloid markers CD14 and CD11b. The macrophage differentiation marker CD14 is also highly expressed by primary human MDM as a response to IL‐10 and distinguishes THP‐1‐derived macrophages from THP‐1 cells that are differentiated into a dendritic cell type with low CD14 expression.^76^, ^77^ Co‐expression of the myeloid CD11b has been reported in primary human muscle tissue macrophages.^78^ Furthermore, the anti‐inflammatory M2‐like markers CD206 and IL‐10 are expressed by resident macrophages of the colon lamina propria.^79^

The tumor‐associated attributes of created macrophages were additionally assessed focusing on marker genes that play a role in neovascularization as well as cellular metabolism and therefore macrophage polarization.

MMPs are important cancer‐related targets promoting vascularization and tumor development.^26^, ^27^ All investigated subtypes of MMPs were upregulated in the M2 macrophage phenotype, which was confirmed in our primary colon cancer TAM data. The metallopeptidase ADAMTS1 was significantly downregulated in all cell types compared to THP‐1 cells and showed the most evident decrease in M2‐like macrophages. Primary TAM scRNA‐Seq data showed no expression of ADAMTS1 among TAMs, which confirms ADAMTS1 playing a minor part in the final macrophage phenotype.

In general, many markers regulating tumor development can be expressed in macrophages and have a dual role, depending on the type of cancer they have been investigated in—such a ABHD5, MGLL, TLR4, or AP‐1.^80^, ^81^, ^82^, ^83^ All of these markers are expressed in the M2 macrophages created by this model and expression was also demonstrated to be present in primary TAMs. This suggests that changes in gene expression due to cell treatment or cell signaling can be assessed. The demonstrated tumor‐associated gene expression patterns are the basis for expression changes in different experimental settings.

A clear similarity of cells created by this model to primary human macrophages and tumor‐associated characteristics of the M2‐like phenotype are to be confirmed by further functional experiments comparing THP‐1 derived macrophages versus primary cells.

In conclusion, M2‐like macrophages created using this 14‐day cell line model of differentiating and polarizing THP‐1 monocytes represent a distinct anti‐inflammatory TAM‐like phenotype. Pro‐ and anti‐inflammatory gene expression patterns, protein expression, and cell surface marker analyses revealed an M2‐like expression profile of macrophages similar to reported gene expression patterns in human primary MDM, with tumor‐associated attributes. The distinct M2‐like macrophage M2a subtype provides the basis to investigate M2‐like macrophage‐mediated mechanisms in vitro in various experimental settings, particularly colon cancer progression.

## AUTHOR CONTRIBUTIONS

**Katharina M. Scheurlen:** Conceptualization (lead); data curation (equal); formal analysis (lead); investigation (lead); methodology (lead); project administration (equal); visualization (equal); writing – original draft (lead); writing – review and editing (equal). **Dylan L. Snook:** Data curation (equal); formal analysis (equal); investigation (equal); software (equal); writing – review and editing (supporting). **Andrew B. Littlefield:** Data curation (equal); formal analysis (supporting); investigation (equal); visualization (equal); writing – review and editing (supporting). **Joan B. George:** Data curation (equal); investigation (supporting); visualization (equal); writing – review and editing (supporting). **Mary A. Parks:** Data curation (equal); formal analysis (supporting); investigation (equal); writing – review and editing (supporting). **Robert J. Beal:** Data curation (equal); investigation (equal); project administration (equal); visualization (equal); writing – review and editing (supporting). **Anne Macleod:** Formal analysis (equal); investigation (equal); methodology (equal); project administration (equal); writing – review and editing (equal). **Daniel W. Riggs:** Formal analysis (equal); software (equal); visualization (equal); writing – review and editing (supporting). **Jeremy T. Gaskins:** Formal analysis (equal); investigation (equal); methodology (lead); software (equal); visualization (equal); writing – original draft (supporting); writing – review and editing (supporting). **Julia Chariker:** Conceptualization (equal); formal analysis (lead); investigation (equal); methodology (equal); software (equal); validation (equal); visualization (equal); writing – original draft (supporting); writing – review and editing (supporting). **Eric C. Rouchka:** Conceptualization (equal); formal analysis (equal); investigation (equal); project administration (equal); supervision (equal); validation (equal); visualization (equal). **Susan Galandiuk:** Conceptualization (equal); data curation (equal); formal analysis (equal); funding acquisition (equal); investigation (equal); methodology (equal); resources (lead); supervision (lead); validation (lead); writing – review and editing (equal).

## FUNDING INFORMATION

This work is supported by the John W. Price and Barbara Thruston Atwood Price Trust and the University of Louisville Cancer Education Program (National Cancer Institute award R25‐CA134283 [ABL and JBG]).

## CONFLICT OF INTEREST STATEMENT

The authors have no conflict of interest to declare.

## ETHICS APPROVAL

The study protocols were approved by the Institutional Review Board at the University of Louisville (IRB number 20.0658). All patient data were obtained from publicly available data sources as described in the manuscript.

## Supporting information


Data S1.

## Data Availability

The data that support the findings are included in the manuscript. Primary human sequencing data are openly available as described in Section 2.

## References

[1] Ginhoux F , Guilliams M . Tissue‐resident macrophage ontogeny and homeostasis. Immunity. 2016;44(3):439‐449. doi:10.1016/j.immuni.2016.02.024 26982352

[2] Gordon S , Pluddemann A . Tissue macrophages: heterogeneity and functions. BMC Biol. 2017;15(1):53. doi:10.1186/s12915-017-0392-4 28662662 PMC5492929

[3] Dantzer R , O'Connor JC , Freund GG , Johnson RW , Kelley KW . From inflammation to sickness and depression: when the immune system subjugates the brain. Nat Rev Neurosci. 2008;9(1):46‐56. doi:10.1038/nrn2297 18073775 PMC2919277

[4] Genin M , Clement F , Fattaccioli A , Raes M , Michiels C . M1 and M2 macrophages derived from THP‐1 cells differentially modulate the response of cancer cells to etoposide. BMC Cancer. 2015;15:577. doi:10.1186/s12885-015-1546-9 26253167 PMC4545815

[5] Italiani P , Boraschi D . From monocytes to M1/M2 macrophages: phenotypical vs. functional differentiation. Front Immunol. 2014;5:514. doi:10.3389/fimmu.2014.00514 25368618 PMC4201108

[6] Lund ME , To J , O'Brien BA , Donnelly S . The choice of phorbol 12‐myristate 13‐acetate differentiation protocol influences the response of THP‐1 macrophages to a pro‐inflammatory stimulus. J Immunol Methods. 2016;430:64‐70. doi:10.1016/j.jim.2016.01.012 26826276

[7] Maess MB , Wittig B , Cignarella A , Lorkowski S . Reduced PMA enhances the responsiveness of transfected THP‐1 macrophages to polarizing stimuli. J Immunol Methods. 2014;402(1–2):76‐81. Epub 2013/11/26. doi:10.1016/j.jim.2013.11.006 24269601

[8] Staples KJ , Smallie T , Williams LM , et al. IL‐10 induces IL‐10 in primary human monocyte‐derived macrophages via the transcription factor Stat3. J Immunol. 2007;178(8):4779‐4785. doi:10.4049/jimmunol.178.8.4779 17404258

[9] Wynn TA , Chawla A , Pollard JW . Macrophage biology in development, homeostasis and disease. Nature. 2013;496(7446):445‐455. doi:10.1038/nature12034 23619691 PMC3725458

[10] Stremmel C , Schuchert R , Wagner F , et al. Yolk sac macrophage progenitors traffic to the embryo during defined stages of development. Nat Commun. 2018;9(1):75. doi:10.1038/s41467-017-02492-2 29311541 PMC5758709

[11] Orecchioni M , Ghosheh Y , Pramod AB , Ley K . Macrophage polarization: different gene signatures in M1(LPS+) vs. classically and M2(LPS‐) vs. alternatively activated macrophages. Front Immunol. 2019;10:1084. doi:10.3389/fimmu.2019.01084 31178859 PMC6543837

[12] Lavin Y , Mortha A , Rahman A , Merad M . Regulation of macrophage development and function in peripheral tissues. Nat Rev Immunol. 2015;15(12):731‐744. doi:10.1038/nri3920 26603899 PMC4706379

[13] Bosshart H , Heinzelmann M . THP‐1 cells as a model for human monocytes. Ann Transl Med. 2016;4(21):438. doi:10.21037/atm.2016.08.53 27942529 PMC5124613

[14] American_Type_Culture_Collection (ATCC). THP‐1 (ATCC®TIB‐202™). 2023. Accessed June 2, 2023. https://wwwatccorg/products/all/TIB‐202aspx#generalinformation

[15] Liu SX , Gustafson HH , Jackson DL , Pun SH , Trapnell C . Trajectory analysis quantifies transcriptional plasticity during macrophage polarization. Sci Rep. 2020;10(1):12273. doi:10.1038/s41598-020-68766-w 32703960 PMC7378057

[16] Baxter EW , Graham AE , Re NA , et al. Standardized protocols for differentiation of THP‐1 cells to macrophages with distinct M(IFNgamma+LPS), M(IL‐4) and M(IL‐10) phenotypes. J Immunol Methods. 2020;478:112721. doi:10.1016/j.jim.2019.112721 32033786

[17] Starr T , Bauler TJ , Malik‐Kale P , Steele‐Mortimer O . The phorbol 12‐myristate‐13‐acetate differentiation protocol is critical to the interaction of THP‐1 macrophages with Salmonella Typhimurium. PLoS One. 2018;13(3):e0193601. doi:10.1371/journal.pone.0193601 29538403 PMC5851575

[18] Surdziel E , Clay I , Nigsch F , et al. Multidimensional pooled shRNA screens in human THP‐1 cells identify candidate modulators of macrophage polarization. PLoS One. 2017;12(8):e0183679. doi:10.1371/journal.pone.0183679 28837623 PMC5570424

[19] Li Y , Mohammad RM . al‐Katib a, Varterasian ML, Chen B. Bryostatin 1 (bryo1)‐induced monocytic differentiation in THP‐1 human leukemia cells is associated with enhanced c‐fyn tyrosine kinase and M‐CSF receptors. Leuk Res. 1997;21(5):391‐397. doi:10.1016/s0145-2126(96)00078-1 9225065

[20] Smith SR , Schaaf K , Rajabalee N , et al. The phosphatase PPM1A controls monocyte‐to‐macrophage differentiation. Sci Rep. 2018;8(1):902. doi:10.1038/s41598-017-18832-7 29343725 PMC5772551

[21] Shapouri‐Moghaddam A , Mohammadian S , Vazini H , et al. Macrophage plasticity, polarization, and function in health and disease. J Cell Physiol. 2018;233(9):6425‐6440. doi:10.1002/jcp.26429 PMC13482560 29319160

[22] Mosser DM , Edwards JP . Exploring the full spectrum of macrophage activation. Nat Rev Immunol. 2008;8(12):958‐969. doi:10.1038/nri2448 19029990 PMC2724991

[23] Zhang Q , Wang H , Mao C , et al. Fatty acid oxidation contributes to IL‐1beta secretion in M2 macrophages and promotes macrophage‐mediated tumor cell migration. Mol Immunol. 2018;94:27‐35. doi:10.1016/j.molimm.2017.12.011 29248877 PMC5801116

[24] Fu XL , Duan W , Su CY , et al. Interleukin 6 induces M2 macrophage differentiation by STAT3 activation that correlates with gastric cancer progression. Cancer Immunol Immunother. 2017;66(12):1597‐1608. doi:10.1007/s00262-017-2052-5 28828629 PMC11028627

[25] Ge Z , Ding S . The crosstalk between tumor‐associated macrophages (TAMs) and tumor cells and the corresponding targeted therapy. Front Oncol. 2020;10:590941. doi:10.3389/fonc.2020.590941 33224886 PMC7670061

[26] Chen Y , Song Y , Du W , Gong L , Chang H , Zou Z . Tumor‐associated macrophages: an accomplice in solid tumor progression. J Biomed Sci. 2019;26(1):78. doi:10.1186/s12929-019-0568-z 31629410 PMC6800990

[27] Tan Ide A , Ricciardelli C , Russell DL . The metalloproteinase ADAMTS1: a comprehensive review of its role in tumorigenic and metastatic pathways. Int J Cancer. 2013;133(10):2263‐2276. doi:10.1002/ijc.28127 23444028

[28] Hannemann N , Jordan J , Paul S , et al. The AP‐1 transcription factor c‐Jun promotes arthritis by regulating cyclooxygenase‐2 and arginase‐1 expression in macrophages. J Immunol. 2017;198(9):3605‐3614. doi:10.4049/jimmunol.1601330 28298526

[29] Miao H , Ou J , Peng Y , et al. Macrophage ABHD5 promotes colorectal cancer growth by suppressing spermidine production by SRM. Nat Commun. 2016;7:11716. doi:10.1038/ncomms11716 27189574 PMC4873969

[30] Xiang W , Shi R , Kang X , et al. Monoacylglycerol lipase regulates cannabinoid receptor 2‐dependent macrophage activation and cancer progression. Nat Commun. 2018;9(1):2574. 29968710 10.1038/s41467-018-04999-8 PMC6030061

[31] Qian BZ , Pollard JW . Macrophage diversity enhances tumor progression and metastasis. Cell. 2010;141(1):39‐51. doi:10.1016/j.cell.2010.03.014 20371344 PMC4994190

[32] Scheurlen KM , Billeter AT , O'Brien SJ , Galandiuk S . Metabolic dysfunction and early‐onset colorectal cancer—how macrophages build the bridge. Cancer Med. 2020;9(18):6679‐6693. doi:10.1002/cam4.3315 33624450 PMC7520341

[33] Eum HH , Kwon M , Ryu D , et al. Tumor‐promoting macrophages prevail in malignant ascites of advanced gastric cancer. Exp Mol Med. 2020;52(12):1976‐1988. doi:10.1038/s12276-020-00538-y 33277616 PMC8080575

[34] Chen S , Saeed A , Liu Q , et al. Macrophages in immunoregulation and therapeutics. Signal Transduct Target Ther. 2023;8(1):207. doi:10.1038/s41392-023-01452-1 37211559 PMC10200802

[35] Delgado‐Ramirez Y , Colly V , Gonzalez GV , Leon‐Cabrera S . Signal transducer and activator of transcription 6 as a target in colon cancer therapy. Oncol Lett. 2020;20(1):455‐464. doi:10.3892/ol.2020.11614 32565970 PMC7285805

[36] Maruyama K , Nemoto E , Yamada S . Mechanical regulation of macrophage function—cyclic tensile force inhibits NLRP3 inflammasome‐dependent IL‐1beta secretion in murine macrophages. Inflamm Regen. 2019;39:3. doi:10.1186/s41232-019-0092-2 30774738 PMC6367847

[37] Gatto F , Cagliani R , Catelani T , et al. PMA‐induced THP‐1 macrophage differentiation is not impaired by citrate‐coated platinum nanoparticles. Nanomaterials (Basel). 2017;7(10):332. doi:10.3390/nano7100332 PMC5666497 29039753

[38] Scheurlen KM , Snook DL , Gardner SA , Eichenberger MR , Galandiuk S . Macrophage differentiation and polarization into an M2‐like phenotype using a human monocyte‐like THP‐1 leukemia cell line. J Vis Exp. 2021;174:e62652. doi:10.3791/62652 34398156

[39] Benjamini Y , Drai D , Elmer G , Kafkafi N , Golani I . Controlling the false discovery rate in behavior genetics research. Behav Brain Res. 2001;125(1–2):279‐284. doi:10.1016/s0166-4328(01)00297-2 11682119

[40] Bland JM , Altman DG . Multiple significance tests: the Bonferroni method. BMJ. 1995;310(6973):170. doi:10.1136/bmj.310.6973.170 7833759 PMC2548561

[41] R_Core_Team . R: A language and environment for statistical computing. R Foundation for Statistical Computing; 2020. Accessed February 21, 2023. https://www.R‐project.org

[42] Phillips N . yarrr: A Companion to the e‐Book "YaRrr!: The Pirate's Guide to R", R package version 015. 2017. Accessed August 11, 2021. https://CRAN.R‐project.org/package=yarrr

[43] Edgar R , Domrachev M , Lash AE . Gene expression omnibus: NCBI gene expression and hybridization array data repository. Nucleic Acids Res. 2002;30(1):207‐210. doi:10.1093/nar/30.1.207 11752295 PMC99122

[44] Tsherniak A , Vazquez F , Montgomery PG , et al. Defining a cancer dependency map. Cell. 2017;170(3):564‐576.e16. doi:10.1016/j.cell.2017.06.010 28753430 PMC5667678

[45] Lee HO , Hong Y , Etlioglu HE , et al. Lineage‐dependent gene expression programs influence the immune landscape of colorectal cancer. Nat Genet. 2020;52(6):594‐603. doi:10.1038/s41588-020-0636-z 32451460

[46] Che LH , Liu JW , Huo JP , et al. A single‐cell atlas of liver metastases of colorectal cancer reveals reprogramming of the tumor microenvironment in response to preoperative chemotherapy. Cell Discov. 2021;7(1):80. doi:10.1038/s41421-021-00312-y 34489408 PMC8421363

[47] Li B , Dewey CN . RSEM: accurate transcript quantification from RNA‐Seq data with or without a reference genome. BMC Bioinformat. 2011;12:323. doi:10.1186/1471-2105-12-323 PMC3163565 21816040

[48] Love M , Anders S , Huber W . Differential analysis of count data–the DESeq2 package. Genome Biol. 2014;15(10):1186.

[49] Hao Y , Hao S , Andersen‐Nissen E , et al. Integrated analysis of multimodal single‐cell data. Cell. 2021;184(13):3573‐3587.e29. doi:10.1016/j.cell.2021.04.048 34062119 PMC8238499

[50] Le T , Phan T , Pham M , et al. BBrowser: making single‐cell data easily accessible. *bioRxiv* 2020. doi:10.1101/2020.12.11.414136

[51] Aras S , Zaidi MR . TAMeless traitors: macrophages in cancer progression and metastasis. Br J Cancer. 2017;117(11):1583‐1591. doi:10.1038/bjc.2017.356 29065107 PMC5729447

[52] Lin Y , Xu J , Lan H . Tumor‐associated macrophages in tumor metastasis: biological roles and clinical therapeutic applications. J Hematol Oncol. 2019;12(1):76. doi:10.1186/s13045-019-0760-3 31300030 PMC6626377

[53] Kaur G , Dufour JM . Cell lines: valuable tools or useless artifacts. Spermatogenesis. 2012;2(1):1‐5. doi:10.4161/spmg.19885 22553484 PMC3341241

[54] NIH Central Resource for Grants and Funding Information . Enhancing reproducibility through rigor and transparency. Accessed May 21, 2023. https://grantsnihgov/policy/reproducibility/indexhtm 2023.

[55] Richter M , Piwocka O , Musielak M , Piotrowski I , Suchorska WM , Trzeciak T . From donor to the lab: a fascinating journey of primary cell lines. Front Cell Dev Biol. 2021;9:711381. doi:10.3389/fcell.2021.711381 34395440 PMC8356673

[56] Gillooly JF , Hayward A , Hou C , Burleigh JG . Explaining differences in the lifespan and replicative capacity of cells: a general model and comparative analysis of vertebrates. Proc Biol Sci. 2012;279(1744):3976‐3980. doi:10.1098/rspb.2012.1129 22810428 PMC3427577

[57] Capes‐Davis A , Theodosopoulos G , Atkin I , et al. Check your cultures! A list of cross‐contaminated or misidentified cell lines. Int J Cancer. 2010;127(1):1‐8. doi:10.1002/ijc.25242 20143388

[58] Fleckenstein E , Uphoff CC , Drexler HG . Effective treatment of mycoplasma contamination in cell lines with enrofloxacin (Baytril). Leukemia. 1994;8(8):1424‐1434. 7520103

[59] Zhang Q , Sioud M . Tumor‐associated macrophage subsets: shaping polarization and targeting. Int J Mol Sci. 2023;24(8):7493. doi:10.3390/ijms24087493 PMC10138703 37108657

[60] Cui D , Yuan W , Chen C , Han R . Identification of colorectal cancer‐associated macrophage biomarkers by integrated bioinformatic analysis. Int J Clin Exp Pathol. 2021;14(1):1‐8. 33532018 PMC7847498

[61] Hollmen M , Figueiredo CR , Jalkanen S . New tools to prevent cancer growth and spread: a ‘Clever’ approach. Br J Cancer. 2020;123(4):501‐509. doi:10.1038/s41416-020-0953-0 32595212 PMC7434904

[62] Metzger R , Winter L , Bouznad N , et al. CCL17 promotes colitis‐associated tumorigenesis dependent on the microbiota. J Immunol. 2022;209(11):2227‐2238. doi:10.4049/jimmunol.2100867 36426975

[63] Kim SM , Kim EM , Ji KY , et al. TREM2 acts as a tumor suppressor in colorectal carcinoma through Wnt1/beta‐catenin and Erk signaling. Cancers (Basel). 2019;11(9):1315. doi:10.3390/cancers11091315 PMC6770495 31489935

[64] Sun H , Tang C , Chung SH , et al. Blocking DCIR mitigates colitis and prevents colorectal tumors by enhancing the GM‐CSF‐STAT5 pathway. Cell Rep. 2022;40(5):111158. doi:10.1016/j.celrep.2022.111158 35926458

[65] Epelman S , Lavine KJ , Randolph GJ . Origin and functions of tissue macrophages. Immunity. 2014;41(1):21‐35. doi:10.1016/j.immuni.2014.06.013 25035951 PMC4470379

[66] Tedesco S , De Majo F , Kim J , et al. Convenience versus biological significance: are PMA‐differentiated THP‐1 cells a reliable substitute for blood‐derived macrophages when studying in vitro polarization? Front Pharmacol. 2018;9:71. doi:10.3389/fphar.2018.00071 29520230 PMC5826964

[67] Daigneault M , Preston JA , Marriott HM , Whyte MK , Dockrell DH . The identification of markers of macrophage differentiation in PMA‐stimulated THP‐1 cells and monocyte‐derived macrophages. PLoS One. 2010;5(1):e8668. doi:10.1371/journal.pone.0008668 20084270 PMC2800192

[68] Meijer K , Weening D , de Vries MP , Priebe MG , Vonk RJ , Roelofsen H . Quantitative proteomics analyses of activation states of human THP‐1 macrophages. J Proteomics. 2015;128:164‐172. doi:10.1016/j.jprot.2015.07.013 26200757

[69] Padilla A , Keating P , Hartmann JX , Mari F . Effects of alpha‐conotoxin ImI on TNF‐alpha, IL‐8 and TGF‐beta expression by human macrophage‐like cells derived from THP‐1 pre‐monocytic leukemic cells. Sci Rep. 2017;7(1):12742. doi:10.1038/s41598-017-11586-2 28986583 PMC5630575

[70] Zhao YL , Tian PX , Han F , et al. Comparison of the characteristics of macrophages derived from murine spleen, peritoneal cavity, and bone marrow. J Zhejiang Univ Sci B. 2017;18(12):1055‐1063. doi:10.1631/jzus.B1700003 29204985 PMC5742288

[71] Hoffmann E , Dittrich‐Breiholz O , Holtmann H , Kracht M . Multiple control of interleukin‐8 gene expression. J Leukoc Biol. 2002;72(5):847‐855. 12429706

[72] Lewis C , Zhu M , Lieu M , et al. CCL18 as a mediator of the pro‐fibrotic actions of M2 macrophages in the Vessel Wall during hypertension. FASEB J. 2017;31(S1):825.2. doi:10.1096/fasebj.31.1_supplement.825.2

[73] Schraufstatter IU , Zhao M , Khaldoyanidi SK , Discipio RG . The chemokine CCL18 causes maturation of cultured monocytes to macrophages in the M2 spectrum. Immunology. 2012;135(4):287‐298. doi:10.1111/j.1365-2567.2011.03541.x 22117697 PMC3372745

[74] Malhotra P , Haslett P , Sherry B , et al. Increased plasma levels of the TH2 chemokine CCL18 associated with low CD4+ T cell counts in HIV‐1‐infected patients with a suppressed viral load. Sci Rep. 2019;9(1):5963. doi:10.1038/s41598-019-41588-1 30979916 PMC6461658

[75] Mantovani A , Sica A , Sozzani S , Allavena P , Vecchi A , Locati M . The chemokine system in diverse forms of macrophage activation and polarization. Trends Immunol. 2004;25(12):677‐686. doi:10.1016/j.it.2004.09.015 15530839

[76] Krakow S , Crescimone ML , Bartels C , et al. Re‐expression of CD14 in response to a combined IL‐10/TLR stimulus defines monocyte‐derived cells with an immunoregulatory phenotype. Front Immunol. 2019;10:1484. doi:10.3389/fimmu.2019.01484 31316520 PMC6611188

[77] Aldo PB , Craveiro V , Guller S , Mor G . Effect of culture conditions on the phenotype of THP‐1 monocyte cell line. Am J Reprod Immunol. 2013;70(1):80‐86. doi:10.1111/aji.12129 23621670 PMC3703650

[78] Kosmac K , Peck BD , Walton RG , et al. Immunohistochemical identification of human skeletal muscle macrophages. Bio Protoc. 2018;8(12):e2883. doi:10.21769/BioProtoc.2883 PMC6105281 30148186

[79] Rőszer T . Understanding the mysterious M2 macrophage through activation markers and effector mechanisms. Mediators Inflamm. 2015;2015:816460. doi:10.1155/2015/816460 26089604 PMC4452191

[80] Zhang H , Guo W , Zhang F , et al. Monoacylglycerol lipase knockdown inhibits cell proliferation and metastasis in lung adenocarcinoma. Front Oncol. 2020;10:559568. doi:10.3389/fonc.2020.559568 33363004 PMC7756122

[81] Chen G , Zhou G , Lotvola A , Granneman JG , Wang J . ABHD5 suppresses cancer cell anabolism through lipolysis‐dependent activation of the AMPK/mTORC1 pathway. J Biol Chem. 2021;296:100104. doi:10.1074/jbc.RA120.014682 33219129 PMC7949079

[82] Oblak A , Jerala R . Toll‐like receptor 4 activation in cancer progression and therapy. Clin Dev Immunol. 2011;2011:609579. doi:10.1155/2011/609579 22110526 PMC3216292

[83] Eferl R , Wagner EF . AP‐1: a double‐edged sword in tumorigenesis. Nat Rev Cancer. 2003;3(11):859‐868. doi:10.1038/nrc1209 14668816

