# Supplementary material for: Anti‐inflammatory mechanisms in cancer research: Characterization of a distinct M2‐like macrophage model derived from the THP‐1 cell line
Source: Cancer Med. 2023 Nov 30;12(23):21172–87. doi: 10.1002/cam4.6681 (PMC10726891; doi:10.1002/cam4.6681)

**Supplemental information**

**Supplemental Table 1**

| **Cell type** | **Sample size N** | **Data** | **Data Source** |
| --- | --- | --- | --- |
| Cell line (THP1)-derived monocytes | 3 | RNA-sequencing | - GEO† database, Phanstiel et al. 2017, GSE96800, samples GSM2599707 and GSM2599708; - https://depmap.org, DepMap ID:   ACH-000146); |
| Cell line (THP1)-derived M2 macrophages | 3 | RNA-sequencing | - Hudson-Alpha Genome Sequencing Center (Huntsville, AL) |
|  | | | |
| Primary human PBMC‡ derived from colon cancer patients | 3 | Single cell RNA-sequencing | - GEO† database, Li et al. 2021, GSE178318; |
| Primary human colon cancer tissue TAM§ | 3 | Single cell RNA-sequencing | - Single Cell Atlas accession E-MTAB-8410 |

**Genomic datasets listed by origin (cell type), data type and source**

*GEO*† *= Gene Expression Omnibus;*

‡*PBMC = peripheral blood mononuclear cells;*

§*TAM = tumor-associated macrophage*

**Supplemental Table 2**

| **Marker** | **THP-1 vs Young M0** | **THP-1 vs Old M0** | **THP-1 vs M2** | **Young M0 vs Old M0** | **Young M0 vs M2** | **Old M0 vs M2** |
| --- | --- | --- | --- | --- | --- | --- |
| ***CD80*** | 0.000*** | 0.000*** | 0.000*** | 0.000*** | 0.000*** | 0.000*** |
| ***TNFα*** | 0.016* | 0.015* | 0.851 | 0.879 | 0.004** | 0.004** |
| ***IL-1β*** | 0.000*** | 0.000*** | 0.796 | 0.000*** | 0.000*** | 0.000*** |
| ***IL-6*** | 0.245 | 0.201 | 0.042* | 0.679 | 0.201 | 0.245 |
| ***CXCL10*** | 0.000*** | 0.000*** | 0.000*** | 0.000*** | 0.000*** | 0.000*** |
| ***CD206*** | 0.000*** | 0.000*** | 0.000*** | 0.000*** | 0.000*** | 0.000*** |
| ***IL-10*** | 0.000*** | 0.000*** | 0.000*** | 0.000*** | 0.000*** | 0.000*** |
| ***IL-8*** | 0.000*** | 0.001*** | 0.923 | 0.000*** | 0.000*** | 0.000*** |
| ***CCL18*** | 0.000*** | 0.000*** | 0.000*** | 0.000*** | 0.000*** | 0.000*** |
| ***CCL22*** | 0.000*** | 0.000*** | 0.000*** | 0.005** | 0.002** | 0.990 |
| ***ABHD5*** | 0.624 | 0.448 | 0.001** | 0.124 | 0.000*** | 0.001** |
| ***ADAMSTS1*** | 0.004** | 0.006** | 0.000*** | 0.662 | 0.000*** | 0.000*** |
| ***AP-1*** | 0.046* | 0.335 | 0.120 | 0.120 | 0.000*** | 0.002** |
| ***MGLL*** | 0.000*** | 0.000*** | 0.000*** | 0.268 | 0.000*** | 0.000*** |
| ***MMP2*** | 0.013* | 0.000*** | 0.000*** | 0.000*** | 0.000*** | 0.000*** |
| ***MMP7*** | 0.000*** | 0.000*** | 0.000*** | 0.000*** | 0.000*** | 0.000*** |
| ***MMP9*** | 0.000*** | 0.000*** | 0.000*** | 0.000*** | 0.000*** | 0.000*** |
| ***MMP12*** | 0.000*** | 0.000*** | 0.000*** | 0.000*** | 0.061 | 0.000*** |
| ***TLR4*** | 0.052 | 0.210 | 0.103 | 0.210 | 0.418 | 0.418 |
|  | FDR corrected p-values for all pairwise comparisons. p<0.05 indicates a significant pairwise comparison.  *p<0.05  ** p≤0.005  *** p≤0.001 | | | | | |

**Corrected p-values for all pairwise comparisons of gene**

**expression between cell types.**

p<0.05 indicates a significant pairwise comparison

*CD = cluster of differentiation; TNFα = tumor necrosis factor α; IL = interleukin; CXCL = C-X-C Motif Chemokine Ligand; CXCL = C-X-C Motif Chemokine Ligand; CCL = C-C Motif Chemokine Ligand; MMP = matrix metalloproteinase; ADAMTS1 = ADAM Metallopeptidase With Thrombospondin Type 1 Motif 1; ABHD5 = alpha/beta-hydrolase domain containing 5; MGLL = monoglyceride lipase; TLR4 = toll like receptor 4; AP-1 = activator protein 1;*

**Supplemental Table 3**

| **Marker** | **Mean baseline expression** | **Mean log_2_ FC** | **Adjusted p-value** |
| --- | --- | --- | --- |
| ***ITGAM*** | 10,367.7 | 5.2 | <0.001*** |
| ***STAB1*** | 3,782.8 | 11.7 | <0.001*** |
| ***CD209*** | 8,348.5 | 6.9 | <0.001*** |
| ***CLEC4A*** | 945.8 | 10.5 | <0.001*** |
| ***IGF1*** | 1,819.4 | 7.9 | <0.001*** |
| ***IL1R1*** | 264.2 | 3.2 | <0.001*** |
| ***CHI3L1*** | 32,803.7 | -1.1 | <0.001*** |
| ***CHI3L2*** | 22.7 | 1.8 | 0.125 |
| ***IL27RA*** | 394.2 | 2.1 | <0.001*** |
| ***TREM2*** | 3,708.5 | 3.7 | <0.001*** |
| ***PTPRC*** | 12,251.0 | 1.7 | <0.001*** |
| ***F8A3*** | 127.6 | 0.9 | 0.238 |
| ***ARG1*** | 1.2 | -2.4 | n/a |
| ***CCL17*** | 2.2 | 3.0 | 0.140 |
| ***CCL1*** | 0.6 | 1.5 | n/a |

FDR corrected p-values for all pairwise comparisons. p<0.05 indicates a significant pairwise comparison.

*p<0.05

**p≤0.005

*** p≤0.001

**Corrected p-values for all pairwise comparisons of gene**

**expression between cell line monocytes (THP-1, N=3) and cell line-derived TAM-like M2 macrophages (N=3).**

p <0.05 indicates a significant pairwise comparison

p = n/a indicates a low mean normalized count, in which no adjusted p-value can be calculated.

*ITGAM = integrin alpha subunit M; STAB1 = stabilin 1; CD = cluster of differentiation; CLEC4A = C-type lectin domain family 4 member A; IGF1 = insulin like growth factor 1; IL1R1 = interleukin 1 receptor type 1; CHI3L = chitinase 3 like; IL27RA = interleukin 27 receptor subunit alpha; TREM2 = triggering receptor expressed on myeloid cells 2; PTPRC = protein tyrosine phosphatase receptor type C; F8A3 = coagulation factor VIII Associated 3; ARG1 = arginase 1; CCL = C-C motif chemokine ligand;*

**Supplemental Table 4**

| **Marker** | **Mean log_2_ FC** | **Percentage of monocytes** | **Percentage of macrophages** | **Adjusted**  **p-value** |
| --- | --- | --- | --- | --- |
| ***CD80*** | 0.1 | 0.2 | 4.1 | <0.001*** |
| ***CD86*** | 0.3 | 30.4 | 45.3 | <0.001*** |
| ***TNFα*** | -0.9 | 24.7 | 5.1 | <0.001*** |
| ***IL-1β*** | -0.8 | 74.7 | 34.8 | <0.001*** |
| ***IL-6*** | 0.3 | 1.9 | 5.8 | 0.008** |
| ***CXCL10*** | 0.1 | 2.7 | 2.7 | 1 |
| ***CD206*** | 1.6 | 0.4 | 43.0 | <0.001*** |
| ***IL-10*** | 0.4 | 1.3 | 10.2 | <0.001*** |
| ***IL-8*** | 0.5 | 84.1 | 60.5 | <0.001*** |
| ***CCL18*** | 1.3 | 0.1 | 19.5 | <0.001*** |
| ***CCL22*** | 0 | 0 | 0.6 | 1 |
| ***MMP2*** | 0.2 | 0 | 9.1 | <0.001*** |
| ***MMP7*** | 0.4 | 0 | 6.1 | <0.001*** |
| ***MMP9*** | 1.1 | 0.6 | 19.1 | <0.001*** |
| ***MMP12*** | 2.2 | 0 | 13.9 | <0.001*** |
| ***ADAMTS1*** | 0 | 0 | 0 | 1 |
| ***ABHD5*** | 0.1 | 19.2 | 26.1 | 1 |
| ***MGLL*** | 0.3 | 0.9 | 13.3 | <0.001*** |
| ***TLR4*** | 0.2 | 13.6 | 24.8 | <0.001*** |
| ***AP-1*** | -1.3 | 87.4 | 56.6 | <0.001*** |

FDR corrected p-values for all pairwise comparisons. p<0.05 indicates a significant pairwise comparison.

*p<0.05

**p≤0.005

*** p≤0.001

**RNA-seq primary sample marker expression in peripheral blood monocytes (N=2099) versus colon cancer tissue TAM (N=656)**

*CD = cluster of differentiation; TNFα = tumor necrosis factor α; IL = interleukin; CXCL = C-X-C Motif Chemokine Ligand; CXCL = C-X-C Motif Chemokine Ligand; CCL = C-C Motif Chemokine Ligand; MMP = matrix metalloproteinase; ADAMTS1 = ADAM Metallopeptidase With Thrombospondin Type 1 Motif 1; ABHD5 = alpha/beta-hydrolase domain containing 5; MGLL = monoglyceride lipase; TLR4 = toll like receptor 4; AP-1 = activator protein 1;*

**Legends for Supplemental Figures**

**Supplemental Figure 1:** Overview of experimental design listing cell culture plates and sample sizes of investigated cell line-derived THP-1 and “young M0” cell samples

**Supplemental Figure 2:** Overview of experimental design listing cell culture plates and sample sizes of investigated cell line-derived THP-1 and “young M0” cell samples.

**Supplemental Figure 3:** Correlation of changes in gene expression between primary human monocytes (PBMCs, N=2099) and primary human tumor-associated macrophages identified by Liu et al. (TAM, N=656) and cell line monocytes (THP-1, N=3) and cell line-derived TAM-like M2 macrophages (N=3). Primary human cell data represents single cell RNA-sequencing data, while cell line-derived results are based on bulk RNA-sequencing data. *CD = cluster of differentiation; TNFα = tumor necrosis factor α; IL = interleukin; CXCL = C-X-C Motif Chemokine Ligand; CXCL = C-X-C Motif Chemokine Ligand; CCL = C-C Motif Chemokine Ligand; MMP = matrix metalloproteinase; ADAMTS1 = ADAM Metallopeptidase With Thrombospondin Type 1 Motif 1; ABHD5 = alpha/beta-hydrolase domain containing 5; MGLL = monoglyceride lipase; TLR4 = toll like receptor 4; AP-1 = activator protein 1;*

**Supplemental Figure 1**


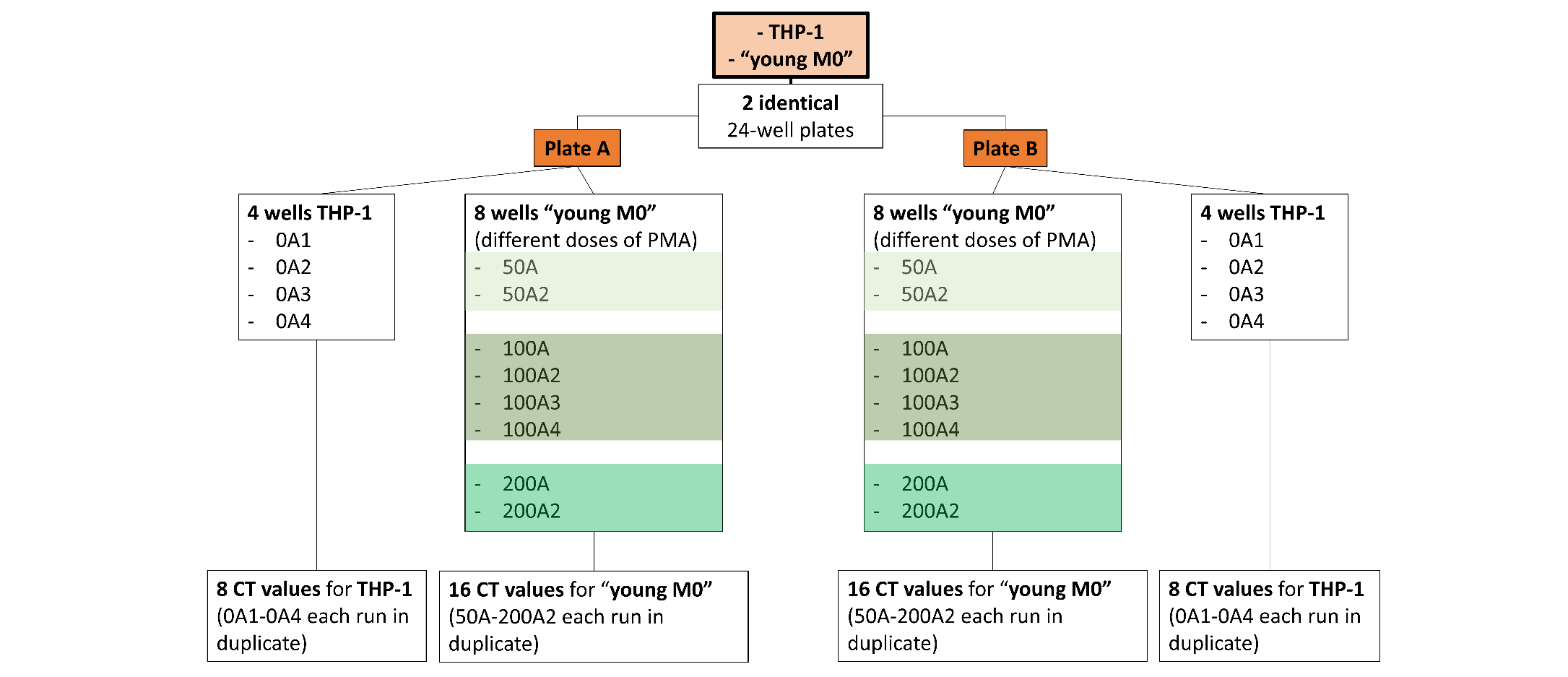


**Supplemental Figure 2**


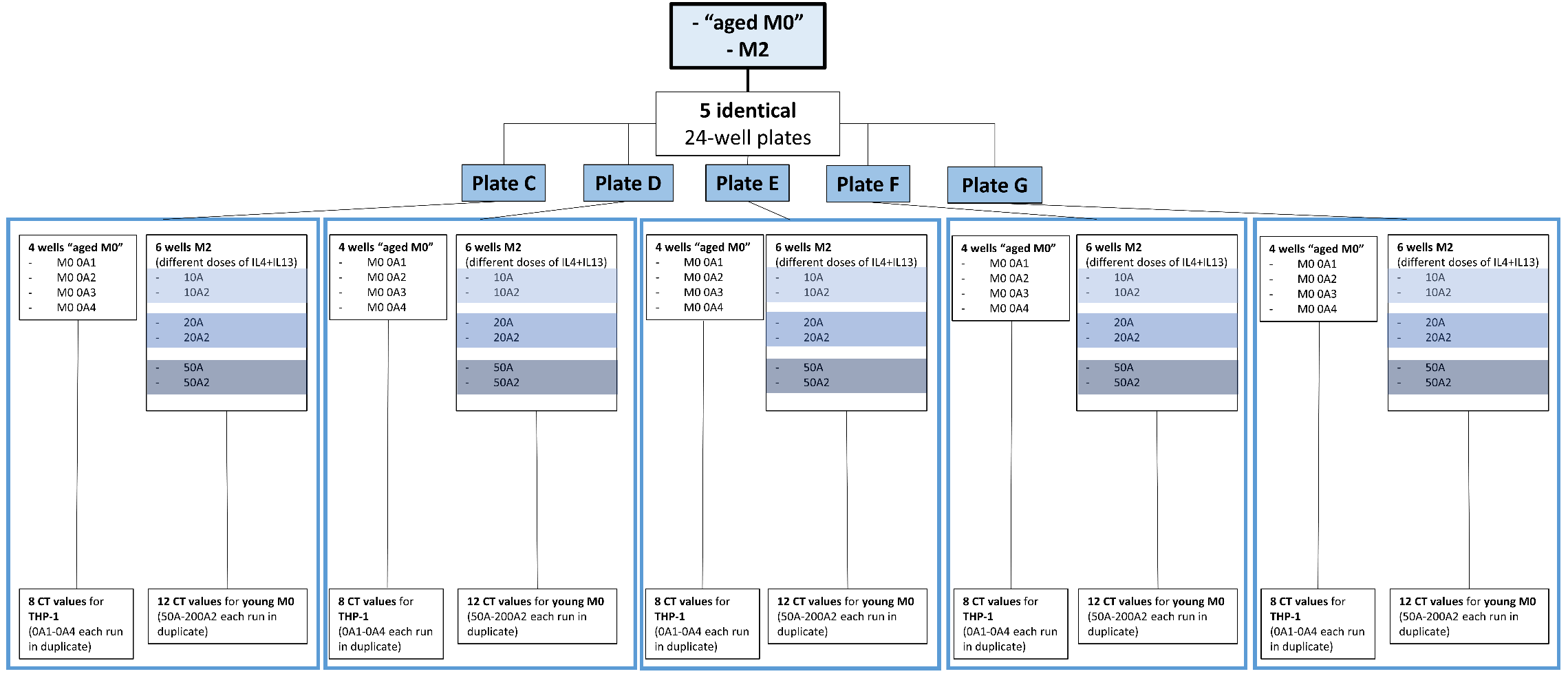


**Supplemental Figure 3**
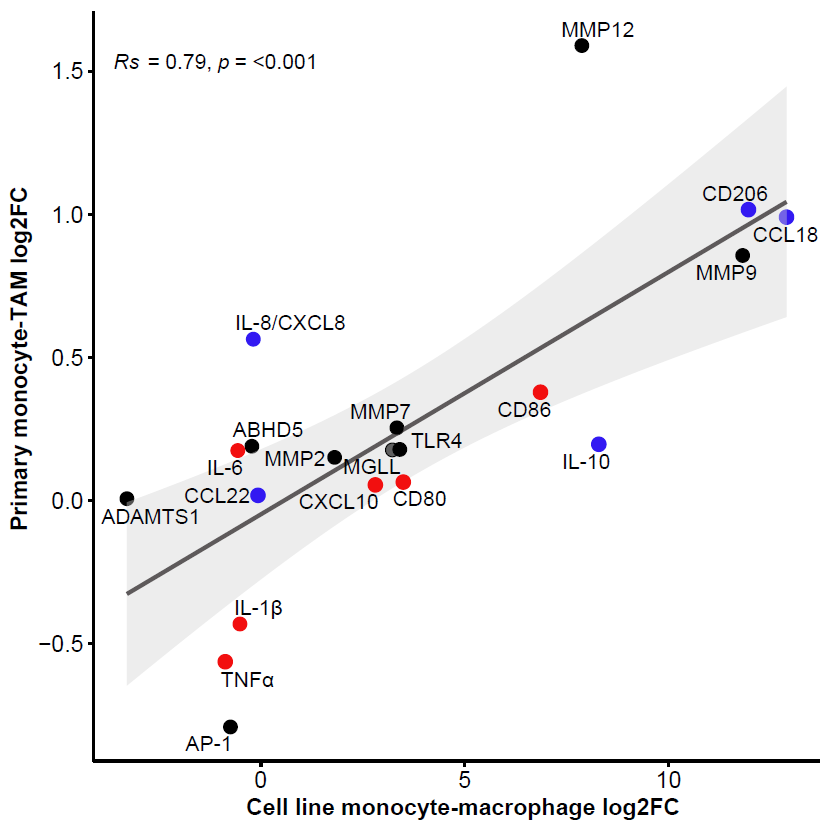

Supplement: Supplementary file 1 — Data S1. [file CAM4-12-21172-s001.docx]
